# Effect of electroacupuncture on hippocampal protein lactylation in a rat model of vascular dementia

**DOI:** 10.3389/fneur.2025.1629474

**Published:** 2025-09-02

**Authors:** Yinghua Chen, Wei Sun, Zhongren Sun, Hongxu Zhao, Tong Wu, Yuanyu Song, Haoyu Wang, Ruiqi Qin, Xiaoqing Su, Junfeng Li, Yue Miao, Xinran Li, Lin Wu

**Affiliations:** ^1^The Fifth Department of Acupuncture, The First Affiliated Hospital of Heilongjiang University of Chinese Medicine, Harbin, China; ^2^Heilongjiang University of Chinese Medicine, Harbin, China

**Keywords:** vascular dementia, electroacupuncture, lactylation, post-translational modification, LC-MS/MS

## Abstract

**Background:**

Vascular dementia (VD) is the only preventable form of dementia-related disease. Electroacupuncture (EA) has been shown to provide significant benefits in the treatment of VD. However, the mechanisms through which EA exerts its therapeutic effects remain unclear. Protein lactylation modification (Kla) is a novel type of post-translational modification that has been shown to be involved in various physiological and pathological processes, including inflammation, immunity, and neurodegenerative diseases. This study, utilizing 4D-Fast data-independent acquisition lactylation quantitative proteomics technology, investigated for the first time the effect of EA intervention on protein lactylation in the hippocampal tissue of rats with VD.

**Methods:**

Rats were randomly assigned to three groups: sham surgery (sham), model [four-vessel occlusion (4-VO)], and EA (4-VO + EA). A rat model of VD was established using the four-vessel occlusion (4-VO) method. The 4-VO + EA group underwent EA intervention at the “Shencong” (Ex-HN01) and “Fengchi” (GB 20) acupoints for 21 consecutive days. After behavioral testing, we collected rat tissues for lactylation modification proteomics analysis.

**Results:**

The results indicate that EA enhances learning and memory in rats. Based on lactylation modification proteomics analysis, compared to the sham group, 93 lactylation sites on 76 lactylated proteins were upregulated, whereas 29 lactylation sites on 25 lactylated proteins were downregulated in the 4-VO group. Compared to the 4-VO group, 381 lactylation sites on 250 lactylated proteins were upregulated, whereas 18 lactylation sites on 14 lactylated proteins were downregulated in the 4-VO + EA group. Of these, 12 lactylated proteins, including Vdac3 and Pacsin1, exhibited significant differences in lactylation modification levels between the 4-VO and sham groups. The sites of lactylation of these proteins tend to recover after EA intervention. Functional enrichment and clustering analyses revealed that these proteins were primarily associated with pathways, including the nucleotide-binding and oligomerization domain (NOD)-like receptor signaling pathway, and synaptic vesicle cycle. Importantly, we assessed whether the lactylation modification level of Vdac3 was enhanced following EA intervention.

**Conclusion:**

EA improved cognitive dysfunction in VD rats, and its mechanism may be related to the regulation of protein lactylation modifications in the hippocampal tissue. It involves multiple targets and pathways and may be related to the enhanced level of Vdac3 lactylation modification.

## 1 Introduction

Vascular dementia (VD) is a type of vascular brain tissue damage caused by reduced perfusion in brain regions and cerebrovascular diseases and is commonly characterized by memory decline and cognitive impairment (1). With the global trend of an aging population and the rising incidence of cerebrovascular diseases, it is estimated that by 2050, over 152.8 million people worldwide will have dementia (2). Moreover, VD is the second most common subtype of dementia after Alzheimer’s disease, and its prevalence increases with age, severely affecting the quality of life of patients and heavily burdening families and society (3). Currently, there is no internationally recognized standardized treatment plan for VD. Few clinical trials on new drugs for VD have been published (4), with treatments mainly relying on cholinesterase inhibitors and N-methyl-D-aspartic acid receptor antagonists, slightly improving cognitive function, while long-term use of drugs may cause a series of adverse reactions (5). As the only reversible type of dementia through early intervention (6), exploring the pathogenesis of VD and effective treatment methods has become the key to treating the disease.

Acupuncture and moxibustion are an important part of traditional Chinese medicine, increasingly attracting worldwide attention and becoming one of the internationally recognized complementary and alternative therapies (7). Notably, acupuncture has been shown in multiple randomized trials and systematic reviews to improve the cognitive abilities and daily living skills of patients with VD (8, 9). Furthermore, electroacupuncture (EA) integrates modern electrophysiological technology while retaining the benefits of traditional acupuncture; by stimulating acupuncture points with microcurrents, it enhances the stimulating and therapeutic effects of acupuncture. Moreover, it has been widely used in the treatment of various neurological diseases (10) due to its strong clinical applicability, standardized electrical stimulation, small side effects, and reliable therapeutic effects. Studies have shown that EA can improve the cognitive function of VD patients compared with traditional simple acupuncture (11). The EA mechanisms in improving the cognitive function of VD model rats include improving hippocampal synaptic transmission efficiency and long-term plasticity (12), reducing neuronal apoptosis (13), inhibiting neuroinflammatory response (14), and enhancing cerebrovascular function (15).

Post-translational modifications (PTMs), including acetylation, glycosylation, phosphorylation, ubiquitination, and palmitoylation, are involved in the regulation of diverse biological processes and cellular physiology by influencing protein structure and function (16). A growing body of research indicates that targeted modulation of specific PTMs with small-molecule inhibitors or activators may mitigate the accumulation of misfolded proteins, thereby potentiating neuroprotective outcomes (17). Lysine lactylation (Kla) has been confirmed to exist in various cells and plays a key role in processes such as cell signaling, gene expression regulation, and cellular metabolism (18, 19). Kla was first discovered in 2019 (20); the results showed that lactate promotes lactylation modifications on lysine residues of histones, further confirming that lactylation plays an important regulatory role in immune cells and cancer metabolism. Recently, three isomers of Kla modifications were identified through orthogonal techniques and L-lactylation modification (K_L-la_) was found to be the primary form of lactylation in cellular histones; together, these findings established its central role in cellular responses to glycolysis and the Warburg effect (21).

Existing studies (22, 23) indicate that EA not only improves glucose metabolism and reduces inflammatory markers but also regulates intracellular metabolic states by influencing lactate production and utilization. As a primary energy substrate, lactate in brain tissue not only provides energy but also contributes to cell signaling by modulating post-translational protein modifications (such as lactylation), playing a crucial role in neuroprotection and cognitive recovery. Consequently, the role of lactylation in neurological diseases has garnered increasing attention, particularly in pathological conditions such as Alzheimer’s disease and VD. For example, Miyakawa et al. (24) observed a significant increase in the lactylation level of histone H1 in depressed mice, suggesting a correlation between depressive states and the lactylation of brain proteins induced by neural excitation. Further, Yuan et al. (25) revealed that the “glycolysis-histone lactylation-PKM2” positive feedback loop formed by abnormal glucose metabolism in microglia can lead to an imbalance in microglial homeostasis and neuroinflammation, which affects epigenetic regulation and promotes the development of Alzheimer’s disease. Additionally, Chen et al. (26) studied the effects of lactate produced by exercise on the microglial phenotype and its relationship with learning and memory. They found that lactate produced by exercise promoted a shift in the microglial polarization balance through lactylation, which is instrumental in alleviating neuroinflammation and improving cognitive function.

Acupuncture improves cognitive function in patients with VD (8) and may affect various pathological processes associated with VD, including neuroprotection, the inhibition of apoptosis, amelioration of neuroinflammation, and regulation of glucose metabolism and neurotransmitter levels (27). Preliminary studies indicate that EA at the “Sishencong” and “Fengchi” points effectively improves cognitive function in patients with VD (28) and enhances learning and memory abilities in VD rats, while also inhibiting the release of inflammatory factors (29). However, the effects of EA on protein lactylation in the hippocampal tissues of VD rats remain unclear, and the specific lactylation sites and regulatory mechanisms in VD require further investigation. Therefore, in this study, we conducted 4D-Fast data-independent acquisition (DIA) quantitative proteomic analysis of lactylation modifications in different groups of rats [sham, four-vessel occlusion (4-VO), and 4-VO + EA] for the first time. We identified 8,852 Kla sites among 2,786 lactylated proteins in the rat hippocampal tissue. Compared with the sham group, 122 Kla sites were identified in 101 differentially proteins in the 4-VO group. Additionally, compared with the 4-VO group, 399 Kla sites were identified in 264 differentially expressed proteins in the 4-VO + EA group. Among them, 12 differentially expressed proteins, such as Vdac3 and Pacsin1, exhibited significant alterations in lactylation modification levels following EA intervention and were significantly enriched in some pathways, including the citrate cycle [tricarboxylic acid (TCA) cycle], synaptic vesicle cycle, and nucleotide-binding and oligomerization domain (NOD)-like receptor signaling pathway. Overall, this study presents novel scientific evidence and actionable targets to further investigate the role of lactation modification in the onset and progression of VD as well as the potential therapeutic benefits of EA intervention for this condition.

## 2 Materials and methods

### 2.1 Animals

A total of 35 male specific-pathogen free-grade Sprague–Dawley rats (8 weeks old weighing 260–280 g) were acquired from the Liaoning Provincial Laboratory Animal Resource Center [Animal Production License: SCXK (Liao) 2020-0001]. The rats were housed individually at the Experimental Animal Center of Heilongjiang University of Chinese Medicine in a tranquil setting for a 7-day acclimatization period. The room temperature was 22 ± 2 °C, the relative humidity was between 50 and 60%, and the rats were maintained on a consistent 12-h light–dark cycle. Food and water were available *ad libitum*. On day 7 of acclimatization, the rats were pre-screened in an aquatic maze; specifically, these rats were allowed to swim freely for 60–90 s in the maze without a platform, and their swimming behaviors were observed during this period. In the preliminary test of the Morris water maze, the mice that showed extreme reactions or remained motionless were excluded, leaving 34 rats eligible for further experimentation. Among these, eight were randomly assigned to the sham group, and the remaining 26 underwent surgery to establish the VD model; 16 of the surgeries were successful. The 16 rats were equally divided into the 4-VO and 4-VO + EA groups, comprising eight rats each. This study was approved by the Animal Experiment and Use Ethics Committee of Heilongjiang University of Chinese Medicine (Animal Research Ethics Number: 2022112506), which ensured compliance with the strict ethical standards for experimental animal use.

### 2.2 4-VO surgery

Rats in the surgical group were subjected to the 4-VO method (30) to prepare a VD model. This method allows permanent bilateral occlusion of the vertebral arteries by cauterization at high temperature, thereby inducing persistent chronic cerebral hypoperfusion. Twenty-four hours following the occlusion of vertebral artery, a temporary occlusion of bilateral common carotid artery will be performed by clamping (31). This staged procedure ensures rats undergo a 24-h adaptation period of hypoperfusion, followed by a series of defined periods of transient complete forebrain ischemia and subsequent reperfusion. However, the technique is technically complex and highly invasive, and it results in a relatively low survival rate (32). The rats were anesthetized by intraperitoneal injection of 2% pentobarbital sodium (35 mg/kg) and subsequently fixed in a prone position with their limbs and head secured. Following disinfection, a midline skin incision was made on the nape and blunt dissection of the subcutaneous and muscle tissue was performed to expose the bilateral foramina of the first cervical vertebra. An electrocautery needle (diameter: 0.5 mm) was used to cauterize both foramina, resulting in permanent occlusion of the bilateral vertebral arteries. A suitable amount of penicillin was administered to prevent wound infection, and the incision was sutured. Twenty-four hours after vertebral artery occlusion, the rats were re-anesthetized with an intraperitoneal injection of 2% pentobarbital sodium (35 mg/kg) and were secured in the supine position with the limbs and head fixed. Following disinfection, a midline incision was made on the ventral neck skin and the subcutaneous muscle tissue was subjected to blunt dissection to expose the bilateral common carotid arteries. Utilizing 4-0 silk thread, the common carotid arteries were identified, secured, and gently retracted. Bilateral microvascular clamps were used to simultaneously occlude the left and right common carotid arteries for 5 min, after which reperfusion was allowed for 1 h. This procedure was conducted three times. Post-reperfusion, the incision was closed with sutures, and prophylactic penicillin was administered to prevent infection. Throughout the 4-VO model induction, physiological parameters were closely monitored. Successful induction of the 4-VO model was determined by the following criteria, observed after bilateral common carotid artery occlusion: loss of consciousness, tachypnea and hyperpnea, absence of the righting reflex, loss of the pupillary light reflex, and absence of a response to noxious stimuli. A postoperative intramuscular injection of penicillin (40,000 U) was administered for seven consecutive days to prevent infection. The Morris water maze experiment was conducted 1 week post-surgery for screening purposes, using the average escape latency of the sham group as a reference value. A successful VD model was defined by a criterion of [(average escape latency of the surgical group rats - reference value)/average escape latency of the surgical group rats] >20% (33). During the surgical procedure, one rat died during anesthesia, and five died during surgery and the postoperative recovery period. Additionally, four rats failed to meet the escape latency testing standards in the Morris water maze experiment, resulting in the final successful modeling of 16 rats.

The sham group was anesthetized and subjected to the same surgical procedures as the VD model group, but without electrocoagulation of the bilateral vertebral arteries or occlusion of the bilateral common carotid arteries.

### 2.3 Intervention methods

**Figure 1:**
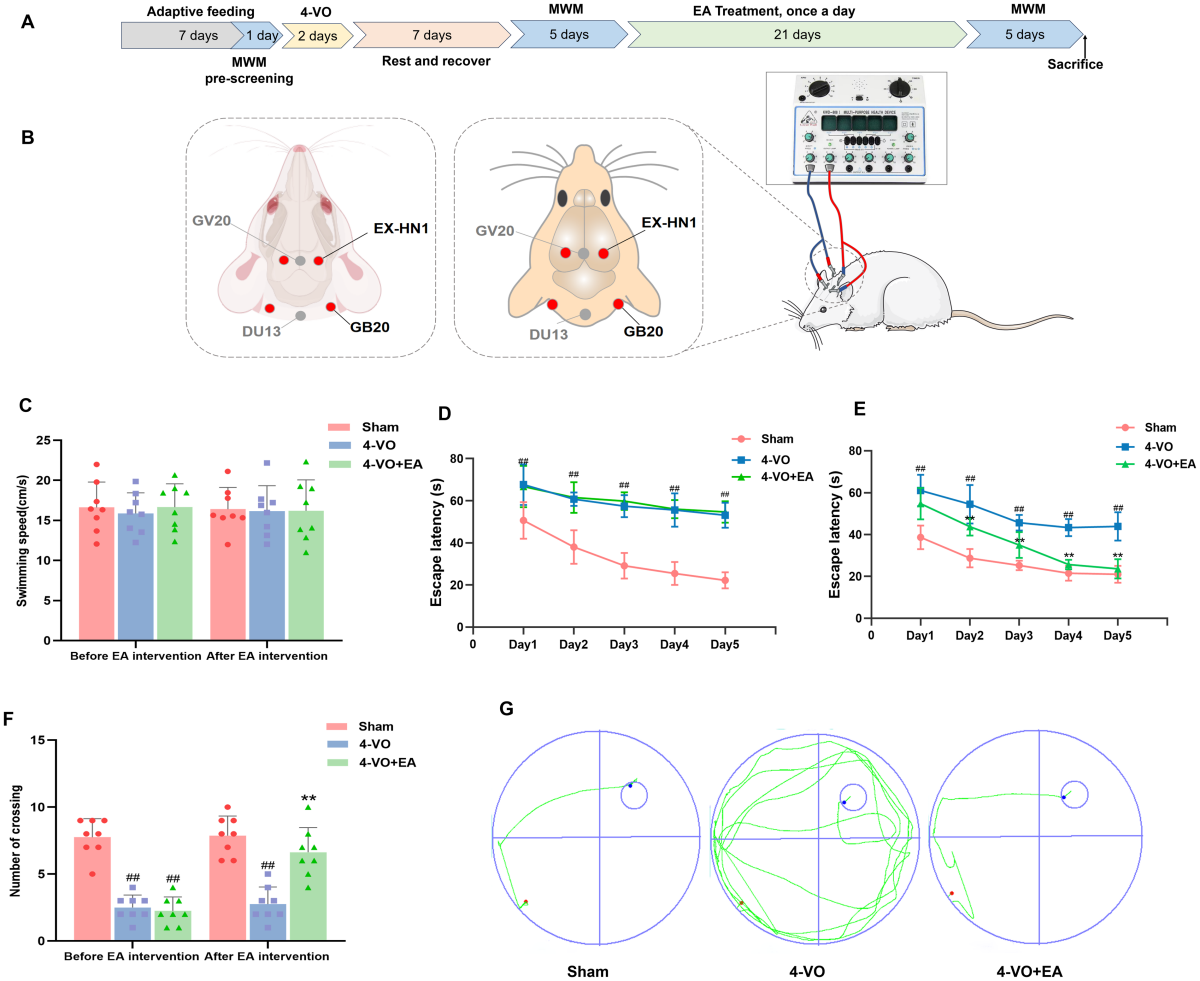
Electroacupuncture enhances the learning and memory capabilities of 4-VO rats. **(A)** The research timeline and the schematic diagram of EA. **(B)** Schematic diagram of acupoint locations and EA stimulation procedures. **(C)** Swimming speed. **(D)** Pre-intervention escape latency. **(E)** Post-intervention escape latency. **(F)** The number of platform crossings. **(G)** Representative trajectory maps for each rat group in the navigation experiment. Compared with the sham surgery group, ^##^*p* < 0.01; compared with the model group, ^**^p < 0.01, *n* = 8. 4-VO, four-vessel occlusion.

EA intervention was initiated 7 days after the animal model was established (Figure 1A). The acupuncture points were bilateral “Sishencong” (Ex-HN01) and bilateral “Fengchi” (GB 20) (34). The rat acupuncture point locations were determined based on the ratio of human to rat skeletal proportions, as outlined in the “Common acupuncture points and localization for experimental animals part 2: rats” (35). The bilateral “Sishencong” (Ex-HN01) points are situated 2 mm lateral to “Baihui” (GV20), whereas the “Fengchi” (GB 20) points are situated 5 mm lateral to “Dazhui” (DU 14) (Figure 1B). Following routine disinfection of the acupoints, disposable acupuncture needles (0.30 mm × 13 mm) were utilized. Left and right “Shencong” (Ex-HN01) points were inserted backward to a depth of approximately 2.5 mm, and bilateral “Fengchi” (GB 20) points were inserted directly to a depth of approximately 5 mm. A KWD-808I EA treatment device was used in this study. One set of electrodes was connected between the ipsilateral “Sishencong” (Ex-HN01) and “Fengchi” (GB 20) points. Continuous wave stimulation was selected at a frequency of 2 Hz and a current intensity of 1 mA. Slight tremors of the rat head were considered as appropriate responses to stimulation. The treatment was administered once daily for 30 min for a total duration of 21 days.

The sham and 4-VO groups were subjected to grasp stimulation. They received the same treatment duration and intensity as the EA group, with no additional treatment.

### 2.4 Morris water maze experiment

The learning and memory abilities of the rats were evaluated using the Morris water maze before and on day 21 after the intervention (36). The experiment consisted of two phases: a navigation test and spatial probe trial. The navigation test was conducted over 5 days, with the platform maintained in the northwest quadrant. The rats were placed in a water maze facing the wall at the same entry point in each quadrant. The escape latency was recorded if the platform was located within 90 s. If the platform was not located within 90 s, the rats were guided to it and allowed to remain there for 10 s, with an escape latency of 90 s recorded. This procedure was repeated for five consecutive days, and the escape latency for each rat was calculated as the average escape latency across the four quadrants on the 5th day. Subsequently, the swimming speeds of each group of rats on day 5 were recorded. Following the navigation test, a spatial probe trial was conducted on the same day. The platform was removed and the rats were placed in the water maze at the southeast quadrant entry point. The number of platform crossings in the target quadrant (previous platform location) was recorded for 90 s. Following each trial, the rats were dried using a towel and returned to their home cages.

### 2.5 Sample collection

After the behavioral tests, each group of rats was anesthetized with an intraperitoneal injection of 2% pentobarbital sodium (35 mg/kg), immediately decapitated on ice, and their brains were rapidly removed. One part was preserved in paraformaldehyde, whereas the other part was quickly dissected from the hippocampal tissue, rapidly frozen in liquid nitrogen, and subsequently stored at −80 °C until testing. Specifically, three rats from each group were subjected to hematoxylin-eosin (HE) staining and lactylation pan-antibody western blot analysis, and five rats from each group were used for ELISAs (enzyme-linked immunosorbent assay) and protein lactylation modification proteomic detection.

### 2.6 HE staining

Fresh brain tissue from each rat group was fixed in 4% paraformaldehyde for 24 h, followed by routine dehydration, paraffin infiltration, and sectioning at a thickness of 4 μm. After drying overnight, the sections were stained with HE. After dewaxing and rehydration, the sections were stained with HE, dehydrated, and mounted with neutral gum. The morphology of the hippocampal tissue was preliminarily examined using an optical microscope (BA310, Motic, China).

### 2.7 ELISA

Each group selected 5 frozen rat hippocampus tissues weighing 20 mg. According to the manufacturers’ instructions for rat TNF-α, IL-1β, and IL-18 ELISA kits (TOPEL02868, TOPEL03514, TOPEL02883; Beijing Biotopped, China), 50 μL of the sequentially diluted standard solutions were added to the standard wells. For sample wells, mixed 10 μL of test sample with 40 μL of sample diluent, then added 100 μL of horseradish peroxidase-conjugated detection antibody. The blank wells did not contain the sample or the enzyme conjugate. Sealed the plate and incubate it at 37 °C for 60 min. Then added the substrate solution, incubated it under dark conditions at 37 °C for 15 min. Terminated the reaction with 50 μL stop solution. Measured the absorbance at 450 nm using a microplate reader, and calculated the concentrations of TNF-α, IL-1β, and IL-18.

### 2.8 Western blotting with a pan anti-lactyllysine antibody

Three rats in each group were administered 15 μg of protein samples, mixed with 4× sample buffer, diluted to 1×, and then combined with the appropriate volume of protein lysis buffer for extraction and quantification. The sample amount was 15 μL, and an equal amount of total cellular protein was separated by electrophoresis (sodium dodecyl sulfate polyacrylamide gel electrophoresis) in a 4 °C environment, with a constant current of 200 mA for 1 h. The protein was then transferred to the nitrocellulose (NC) membrane at a low temperature. The NC membranes were incubated with a 5% skim milk powder blocking solution for 1 h at room temperature, followed by a brief wash step (2–3 min) after removing the blocking solution. The membranes were then incubated with a primary anti-lactyllysine antibody (PTM-1401RM, PTM Bio, China) diluted 1: 150 in an appropriate buffer overnight at 4 °C. Subsequently, the membranes were incubated with the corresponding horseradish peroxidase-conjugated secondary antibody (1: 10,000 dilution, Pierce31460, Thermo, United States) for 1 h at room temperature. Finally, a chemiluminescent horseradish peroxidase substrate (WBKLS0500, Millipore, United States) was applied for 2 min, and the signal was detected using a chemiluminescent imaging system following the manufacturer’s instructions.

### 2.9 Protein extraction

The samples were removed from storage at −80 °C, and an appropriate amount was weighed into a liquid nitrogen pre-cooled mortar. The samples were then thoroughly ground into powder using liquid nitrogen. For each group, four times the volume of lysis buffer (8 M urea, 1% protease inhibitor, 3 μM Trichostatin A, 50 mM Nicotinamide) was added before subjecting the mixture to ultrasonic lysis. The lysates were centrifuged at 12,000 g for 10 min at 4 °C to remove cell debris. The supernatant was transferred to a new centrifuge tube for protein concentration measurements using a bicinchoninic acid assay kit (P0011, Beyotime, China).

### 2.10 Trypsin digestion

Equal amounts of protein from each sample were subjected to enzymatic digestion. Trichloroacetic acid was slowly added to a final concentration of 20%, followed by vortexing and mixing. The mixture was then precipitated at 4 °C for 2 h. The supernatant was removed after centrifugation at 4,500 × g for 5 min, and the precipitate was washed 2–3 times with pre-cooled acetone. After drying the precipitate, a final concentration of 200 mM triethylammonium bicarbonate was added. The precipitate was sonicated to break it up, followed by the addition of trypsin at a ratio of 1: 50 (trypsin: protein, m/m) for overnight digestion. Dithiothreitol was then added to a final concentration of 5 mM, and the solution was incubated at 56 °C for 30 min to reduce disulfide bonds. Subsequently, iodoacetamide was added to a final concentration of 11 mM, and the solution was incubated at room temperature for 15 min in the dark.

### 2.11 Affinity enrichment

The peptides were dissolved in an immunoprecipitation buffer solution containing 100 mM NaCl, 1 mM ethylenediaminetetraacetic acid, 50 mM Tris-HCl, and 0.5% NP-40, pH 8.0. The supernatant was then transferred to pre-washed antibody resin (Anti-L-Lactyl Lysine Antibody, PTM1404, PTM Bio, China) and placed on a rotary shaker at 4 °C, where it was gently shaken and incubated overnight. After incubation, the resin was sequentially washed four times with the immunoprecipitation buffer and twice with deionized water. The resin-bound peptides were subsequently eluted three times using 0.1% trifluoroacetic acid, and the combined eluents were collected and freeze-dried under vacuum. Following extraction and desalting according to the C18 ZipTips protocol, the solution was freeze-dried under vacuum and analyzed using liquid chromatography-mass spectrometry.

### 2.12 Liquid chromatography-mass spectrometry analysis

The peptides were separated using an Easy-nLC1000 UHPLC system following solubilization in liquid chromatography mobile phase A, which consisted of an aqueous solution containing 0.1% formic acid and 2% acetonitrile. Mobile phase B was composed of an acetonitrile-water solution with 0.1% formic acid. The liquid-phase gradient was programmed as follows: 0–18 min, 9–24% B; 18–22 min, 24–35% B; 22–26 min, 35–90% B; and 26–30 min, 90% B. The flow rate was maintained at 450 nL/min. The peptides were separated using a ultra performance liquid chromatography system, ionized in a capillary ion source, and subsequently analyzed using a timsTOF Pro mass spectrometer. The ion source voltage was set to 1.6 kV, and the peptide parent ion and its secondary fragments were detected and analyzed using TOF in the data-independent parallel accumulation serial fragmentation mode. The primary mass spectrometry scan range was set to 100–1,700 m/z, followed by one primary mass spectrometry acquisition and 10 parallel accumulation serial fragmentation mode acquisitions. Secondary mass spectrometry scans were conducted at an interval of 400–1,200 m/z, with a window of 25 m/z each.

### 2.13 Database search

The DIA data for this experiment were searched using the Spectronaut (v 17) search engine with default software parameters. The *R. norvegicus* 10116_PR 20230103. fasta (47,945 sequences) database was used. The digestion mode was set to trypsin/P, with four missed cleavage sites. Carbamidomethyl (C) was used as the fixed modification for cysteine alkylation. Various modifications included oxidation of methionine, oxidation of protein N-terminus acetylation, and lactonization of lysine. An inverse library was added to calculate the false discovery rate owing to random matching, with the false discovery rate for protein, peptide, and peptide-spectrum match identification set to 1%.

### 2.14 Bioinformatic analysis

Gene Ontology (GO) annotations were employed to extract GO IDs for each identified protein using eggnog-mapper software (v5.0.2, http://eggnog5.embl.de/#/app/home) for functional classification analysis. Structural domains were annotated for the identified proteins using the Pfam database.^1^ The Kyoto Encyclopedia of Genes and Genomes (KEGG) pathway database^2^ was used to annotate protein pathways. Functional enrichment of proteins was performed using the GO, KEGG, and Pfam databases for the classification of differentially expressed proteins, KEGG pathway, and protein structural domain enrichment analysis. Cluster analysis was conducted based on the functional enrichment of the differentially expressed modified proteins in various subgroups to explore their potential connections and differences in specific functions, including GO, KEGG pathways, protein structural domains, reactome, and wiki pathways. The database numbers or protein sequences of differentially expressed modifier proteins identified in the comparison group were extracted to obtain the interactions, based on a confidence score >0.7 (high confidence), following comparison with the STRING Protein Interaction Network database.

### 2.15 Immunoprecipitation and western blotting determination of Vdac3 lactylation modification

Hippocampal tissues from three rats per group were homogenized in pre-cooled radioimmunoprecipitation assay lysis buffer and centrifuged at 12,000 g for 15 min at 4 °C to collect the supernatant. One milliliter of total protein extract was pre-cleared with 40 μL of protein A/G agarose beads by rotation at 4 °C for 1 h. Subsequently, the supernatant was incubated with 5 μL of Vdac3 antibody (A04802-2, BosterBio, China) overnight at 4 °C with rotation. Next, 40 μL of fresh protein A/G agarose beads were added and incubated at 4 °C for 2 h. The beads were washed with PBS, and proteins were eluted by adding 2× sodium dodecyl sulfate loading buffer and heating at 95 °C for 5 min. Twenty microliters of sample were loaded, and proteins were transferred to polyvinylidene difluoride (PVDF) membranes at 100 V for 90 min. The membranes were washed with tris-buffered saline with Tween-20 (TBST) for 5 min and blocked with 5% skimmed milk for 1 h. Primary antibody incubation was performed using Vdac3 antibody (1: 1,000 dilution in 5% skimmed milk) for 2 h, followed by three 10-min TBST washes. The membranes were then incubated with horseradish peroxidase (HRP)-labeled secondary antibody (1: 5,000 dilution; M21002, Abmart, China) for 1 h and developed using chemiluminescent HRP substrate (WBKLSO500, Milipore, United States). For subsequent lactylation detection, the PVDF membranes were stripped using membrane regeneration solution (SW3020, Solarbio, China) for 20 min, washed three times with TBST for 10 min each, and blocked with 5% skimmed milk for 1 h. The membranes were then incubated with lactylation antibody (1, 2000 dilution in 5% skimmed milk; PTM-1429RM, PTM Bio, China) for 2 h, followed by three 10-min TBST washes, incubation with HRP-labeled secondary antibody for 1 h, and visualization using chemiluminescent HRP substrate.

### 2.16 Statistical analysis

Statistical analyses were conducted using SPSS 23.0, and GraphPad Prism 9 was used to generate the graphs. All data were subjected to normality and homogeneity of variance testing. Measurement data that followed a normal distribution were presented in the form of mean ± standard deviation. Two-way repeated measures analysis of variance was used to analyze the water maze data, with subgroups treated as between-group factors and different time points as within-group factors. When a significant interaction effect was detected, the least significant difference (LSD) method was used for a simple effect analysis to compare the effects of independent groups. For data that met homogeneity of variance assumptions, one-way analysis of variance (ANOVA) was employed for multi-group comparisons, with pairwise comparisons conducted using Fisher’s least significant difference (LSD) *post hoc* test. For data that violated the homogeneity of variance assumption, Dunnett’s T3 test was applied. The reproducibility of proteomic data was assessed via Pearson correlation analysis. The protein intensity values were log10 transformed to standardize the distribution, and then pairwise Pearson correlation coefficients were calculated between all samples. A *p*-value of less than 0.05 was considered indicative of statistical significance.

## 3 Results

### 3.1 EA improves learning memory in 4-VO rats

As demonstrated in the experimental flowchart and the EA schematic diagram (Figures 1A,B), water maze tests were performed before and after EA treatment. Prior to the intervention, mean swimming speeds revealed no statistically significant differences between rat groups (*p* > 0.05) (Figure 1C), compared with the sham group, the 4-VO and 4-VO + EA groups exhibited a significantly prolonged latency and a markedly reduced number of platform crossings (*p* < 0.01) (Figures 1D,F), indicating successful modeling. Post-intervention, mean swimming speeds revealed no statistically significant differences between rat groups (*p* > 0.05), compared to the sham group, the 4-VO group displayed a significantly prolonged latency and a notably reduced number of platform crossings (*p* < 0.01). Conversely, compared to the 4-VO group, the 4-VO + EA group exhibited a significantly shortened latency and a considerably increased number of platform crossings (*p* < 0.01) (Figures 1E,F), suggesting that EA enhanced the learning and memory abilities of 4-VO rats (Figure 1G). Statistical datasets are detailed in Supplementary File 1.

### 3.2 The effect of EA on the hippocampal tissue pathology, inflammatory mediators, and total protein Kla level in the hippocampal tissue of VD rats

**Figure 2:**
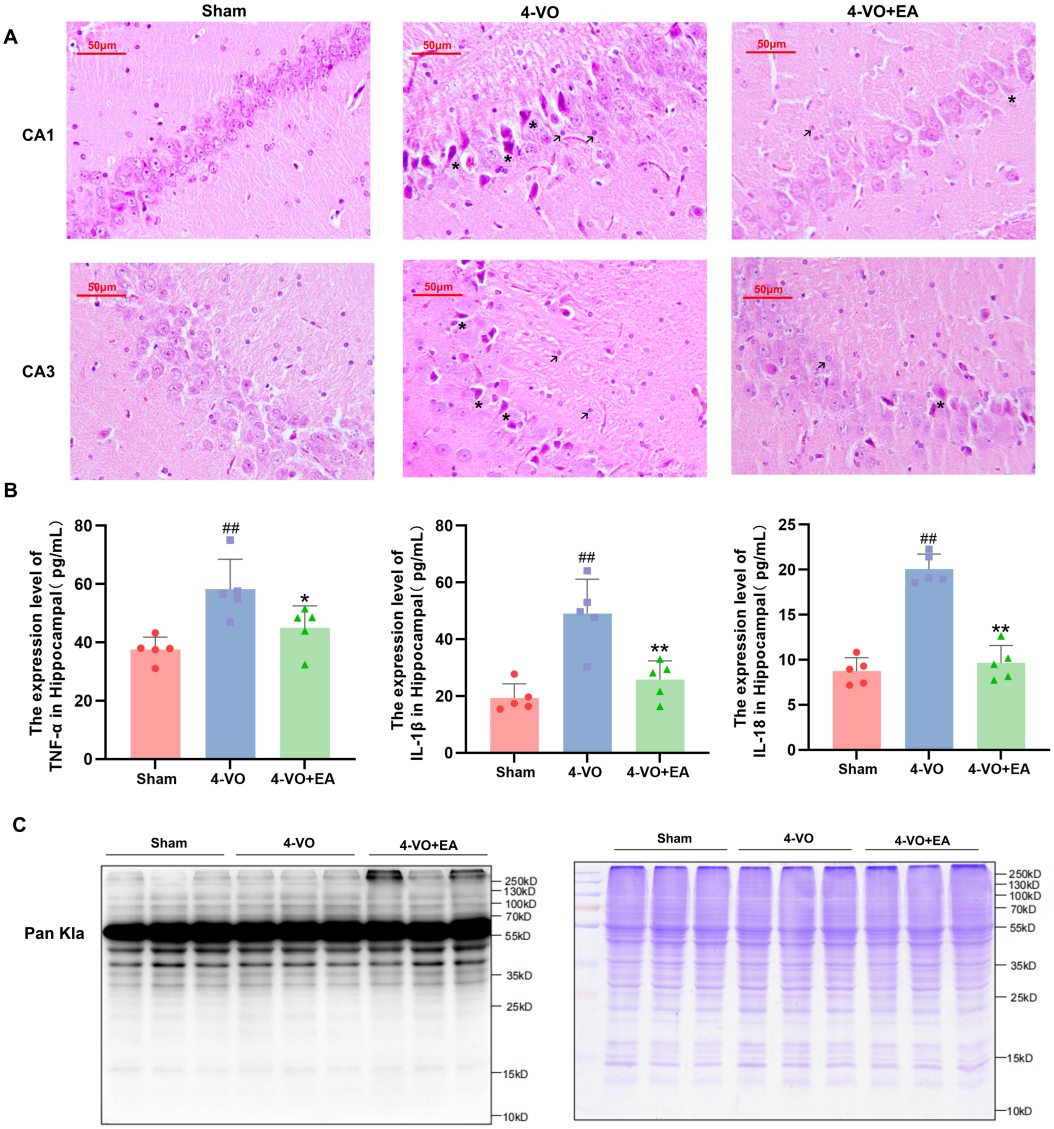
The effect of electroacupuncture on the pathological morphology and total protein Kla level in the hippocampus of VD rats. **(A)** HE staining of hippocampal tissues in each group of rats (*n* = 3), with asterisks marking nuclear pyknosis and apoptotic bodies and arrows marking inflammatory cells. **(B)** Comparative analysis of the levels of TNF-α, IL-1β, and IL-18 in hippocampal tissues of different experimental rat groups. (*n* = 5, compared with the sham group, ^##^*p* < 0.01; compared with the 4-VO group, ^**^p < 0.01). **(C)** Western blot analysis using a pan antibody for lactyllysine was conducted, revealing significant changes in lactyllysine in the hippocampal tissue of VD rats subjected to electroacupuncture. “123” refers to the Sham group, “456” to the 4-VO group, and “789” to the 4-VO + EA group. The loading volume was 15 μg protein/lane primary antibody: anti-lactyllysine antibody (PTM-1401RM; Lot: RL063009; 1:150 dilution), 2nd antibody: Thermo, Pierce, goat anti-rabbit IgG, (H + L), peroxidase conjugated, 31,460, 1:10,000 dilution. Coomassie brilliant blue staining to verify the quality of protein extraction in the samples. 4-VO, four-vessel occlusion; HE, hematoxylin-eosin; EA, electroacupuncture; VD, vascular dementia.

The results of HE staining indicated that, compared with the 4-VO group, the overall morphology of hippocampal neurons in the 4-VO + EA group appeared more regular, the phenomenon of nuclear atrophy was alleviated, the infiltration of inflammatory cells was reduced, and the formation of apoptotic bodies was also decreased. EA improved the pathological morphology of the hippocampal tissue in VD rats to a certain degree (Figure 2A). Compared with the sham group, the levels of TNF-α, IL-1β, and IL-18 in hippocampal tissue were significantly elevated in the 4-VO group (*p* < 0.01). Compared with the 4-VO group, these cytokine levels in both hippocampal tissues were significantly reduced in the 4-VO + EA group (*p* < 0.01) (Figure 2B). To evaluate the effect of EA on the expression profile of Kla protein in hippocampal tissue of VD rats, a Kla polyclonal antibody was used to measure the total protein lactylation levels in nine rats (three per group: sham, 4-VO, and 4-VO + EA) using western blotting. Lactylation profiles visualized via pan-Kla antibody staining revealed preliminary, non-quantitative trends. We observed that in the hippocampal tissue of VD rats treated with EA, the Kla intensity pattern changed (Figure 2C). Detailed statistical datasets and the original images of the WB experiments are provided in Supplementary File 2.

### 3.3 Quantitative proteomic analysis of lactylation modifications in the hippocampal tissue from three groups of rats

**Figure 3:**
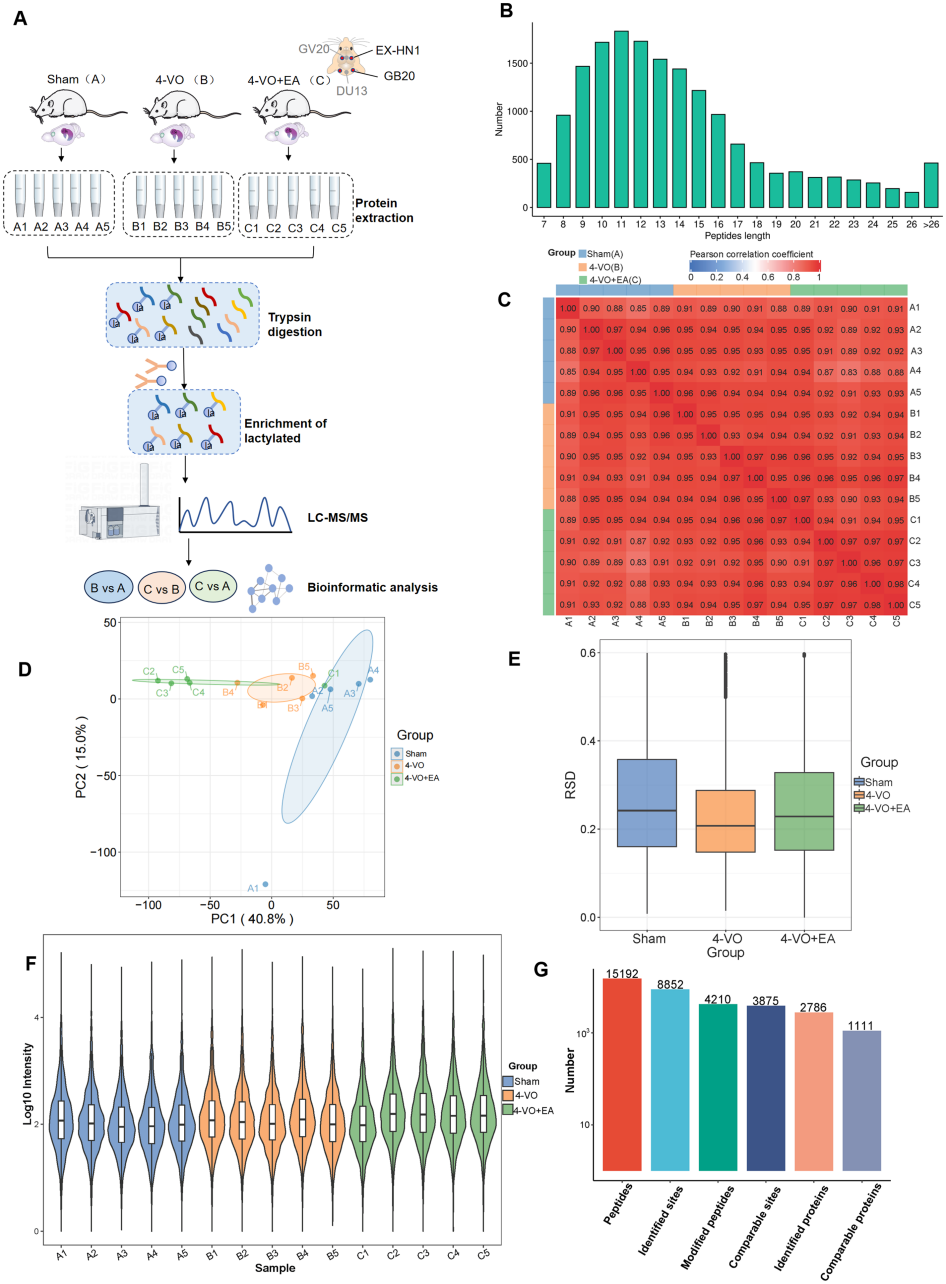
Quantitative proteomic analysis of lactylation modifications in the hippocampal tissues of the three groups. **(A)** The workflow of 4D-Fast DIA lactylation-modified quantitative proteomics analysis of hippocampal tissues in three groups of rats: Sham group, 4-VO group, and 4-VO + EA group (with 5 replicates in each group). The Sham group is abbreviated as Group A, with samples A1–A5; the 4-VO group is abbreviated as Group B, with samples B1–B5; the 4-VO + EA group is abbreviated as Group C, with samples C1–C5. **(B)** Distribution of peptide segment lengths identified using mass spectrometry. **(C)** Heatmap depicting the Pearson correlation coefficient between sample pairs based on the intensity values from all samples. **(D)** Principal component analysis plot derived from the relative quantification values of all samples. **(E)** Box plot showing the relative standard deviation calculated from the relative standard deviation (RSD) of replicate samples within each group. **(F)** Violin plot showing the modification site intensity values for each sample. **(G)** Summary of the identified peptide segments, modification sites, and protein numbers.

4D-Fast DIA lactylation modification quantitative proteomics analysis was conducted to identify Kla in the hippocampal tissues of 15 rats (five in the sham group, five in the 4-VO group, and five in the 4-VO + EA group), with five repetitions per group. The workflow of the study is shown in Figure 3A. The peptide segments identified by mass spectrometry were distributed in 7–20 amino acids, which is in line with the general rules based on trypsin digestion and high-energy collision-induced dissociation. Given the small amount of fragment ions, peptides with five or fewer amino acids cannot produce effective sequence recognition. Moreover, peptides with more than 20 amino acids are not suitable for high-energy collision-induced dissociation due to their high quality and charge number. The length distribution of peptide segments identified by mass spectrometry met the quality control requirements and had credible accuracy (Figure 3B).

The Pearson product–moment correlation coefficient between samples and the intra-group correlation within each group ranges from 0.8 to 1, demonstrating high correlation. Under the context of biological replication, this suggests consistent experimental conditions and treatments (Figure 3C). Moreover, principal component analysis reveals that the three groups of samples are clearly distinguishable (Figure 3D). For the biological and technical replicate samples, we evaluated whether the quantitative results were consistent. The relative standard deviation (RSD) for each group was approximately 0.2, and the overall RSD value was relatively low, indicating good repeatability of the quantitative results (Figure 3E). The violin plot indicates that the sample means align horizontally, reflecting good sample quality (Figure 3F). In conclusion, the samples are deemed to have passed quality control and are suitable for subsequent analysis. Overall, the data met the standards and demonstrated reliable accuracy. Following data filtration, 15,192 peptides and 4,210 modified peptides were identified using a mass spectrometry database search. Among the 2,786 proteins, 8,852 lactylation sites were identified. Additionally, among the 1,111 quantifiable proteins, modification sites contained quantitative information (Figure 3G and Supplementary Table 1).

### 3.4 Identification of lactylation modified proteins in the hippocampal tissue of the three groups of rats

**Figure 4:**
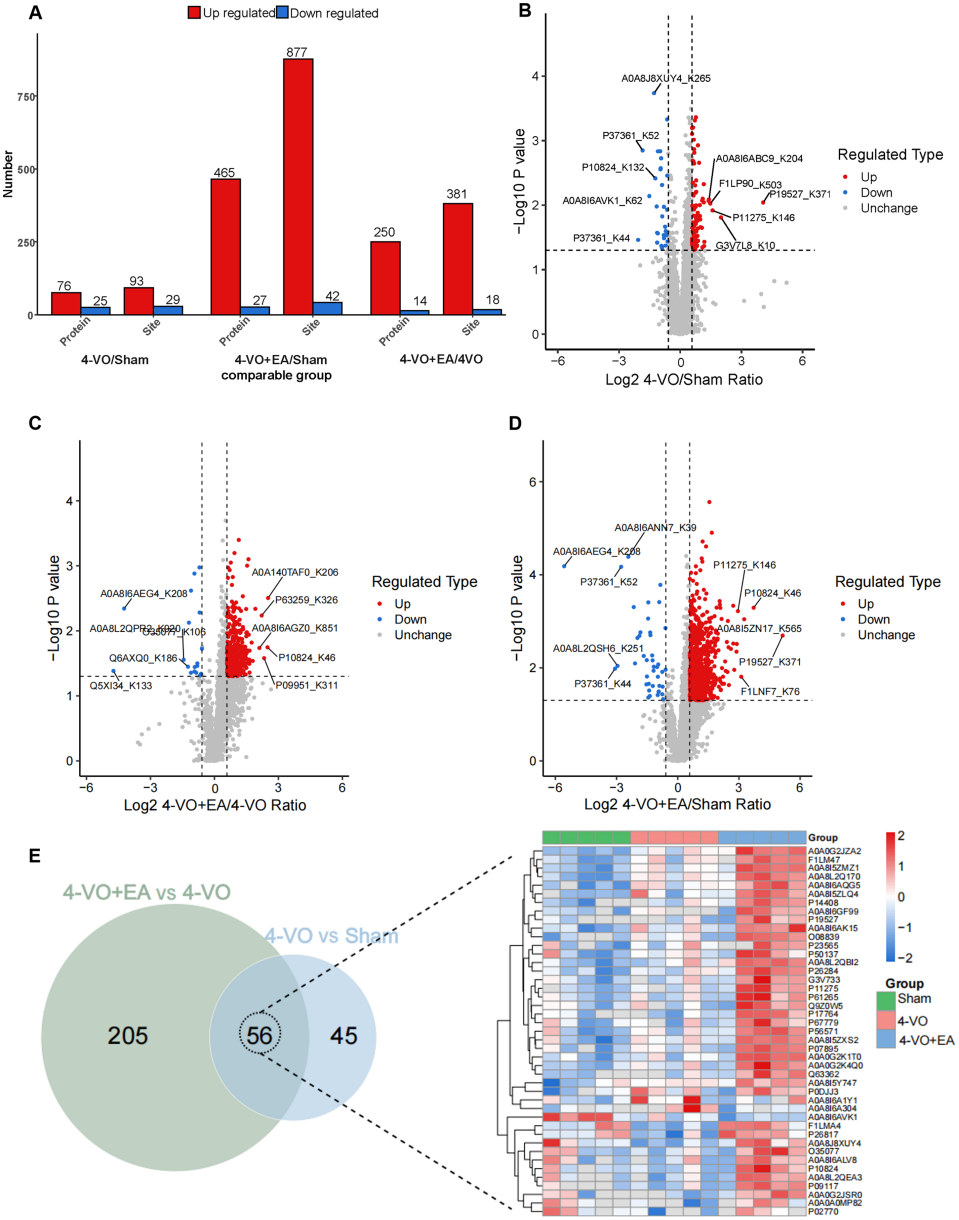
Identification of lactylation modified proteins in the hippocampal tissue of three groups of rats. **(A)** The quantities of proteins that were differentially expressed and the sites that were differentially modified across the three groups are presented. **(B)** A volcano plot illustrating the differential modification sites between the 4-VO and sham groups. **(C)** A volcano plot displaying the differences in modification sites between the 4-VO + EA and 4-VO groups. **(D)** A volcano plot showing the differential modification sites between the 4-VO + EA and sham groups. **(E)** On the left, a Venn diagram depicts the intersection of proteins associated with differential modification sites from comparisons 4-VO + EA vs. 4-VO and 4-VO vs. Sham; on the right, the relative quantification of the 56 intersecting proteins harboring lysine lactylation sites is displayed in a heatmap, showcasing their expression over 15 samples. 4-VO, four-vessel occlusion; EA, electroacupuncture.

The ratio of the mean relative quantification values for the modification sites across the three sample groups was calculated as the fold change. This calculation was performed to determine the relative levels of modification among the three groups. Through the differential analysis mentioned above, a threshold greater than 1.5 was established for significant upregulation, whereas a threshold of less than 1/1.5 was set for significant downregulation when the *p*-value was less than 0.05. This allowed the identification of genes and sites that exhibited significant changes in expression. The results of this analysis are presented in Figure 4A, which shows the number of differentially expressed proteins and modification sites among the three groups. In comparison with the sham group, the 4-VO group exhibited 76 upregulated and 25 downregulated proteins, with 93 upregulated and 29 downregulated sites (Figure 4B). In contrast, the 4-VO + EA group displayed 250 upregulated and 14 downregulated proteins, with 381 upregulated and 18 downregulated sites, compared to the 4-VO group (Figure 4C). Furthermore, compared to the sham group, the 4-VO + EA group had 465 upregulated proteins and 27 downregulated proteins, with 877 upregulated and 42 downregulated sites (Figure 4D and Supplementary Table 2). Collectively, a total of 56 lactylation sites were identified on the differentially expressed proteins in both comparison sets (sham vs. 4-VO; 4-VO vs. 4-VO + EA). Lactylated proteins hosting these sites are presented in the heatmap (Figure 4E). Of these proteins, 12 exhibited significant changes in protein lactylation modification levels between the 4-VO and sham groups (two upregulated and 10 downregulated), which were notably reversed following EA intervention (two downregulated and 10 upregulated). The proteins included A0A0A0MP82 (Hba-a3), A0A0G2JSR0 (Vdac3), A0A8I6A1Y1 (Ogdh), A0A8I6A304 (Basp1), A0A8I6APA7 (Nefh), A0A8J8XUY4 (Mapt), A0A8L2QEA3 (Hsp90ab1), P02770 (Alb), P10824 (Gnai1), P17764 (Acat1), P26817 (Grk2), and Q9Z0W5 (Pacsin1). We speculate that these 12 lactylation-modified proteins may be key targets for EA treatment of VD.

### 3.5 Functional enrichment of lactylation modified proteins in the 4-VO and sham groups

**Figure 5:**
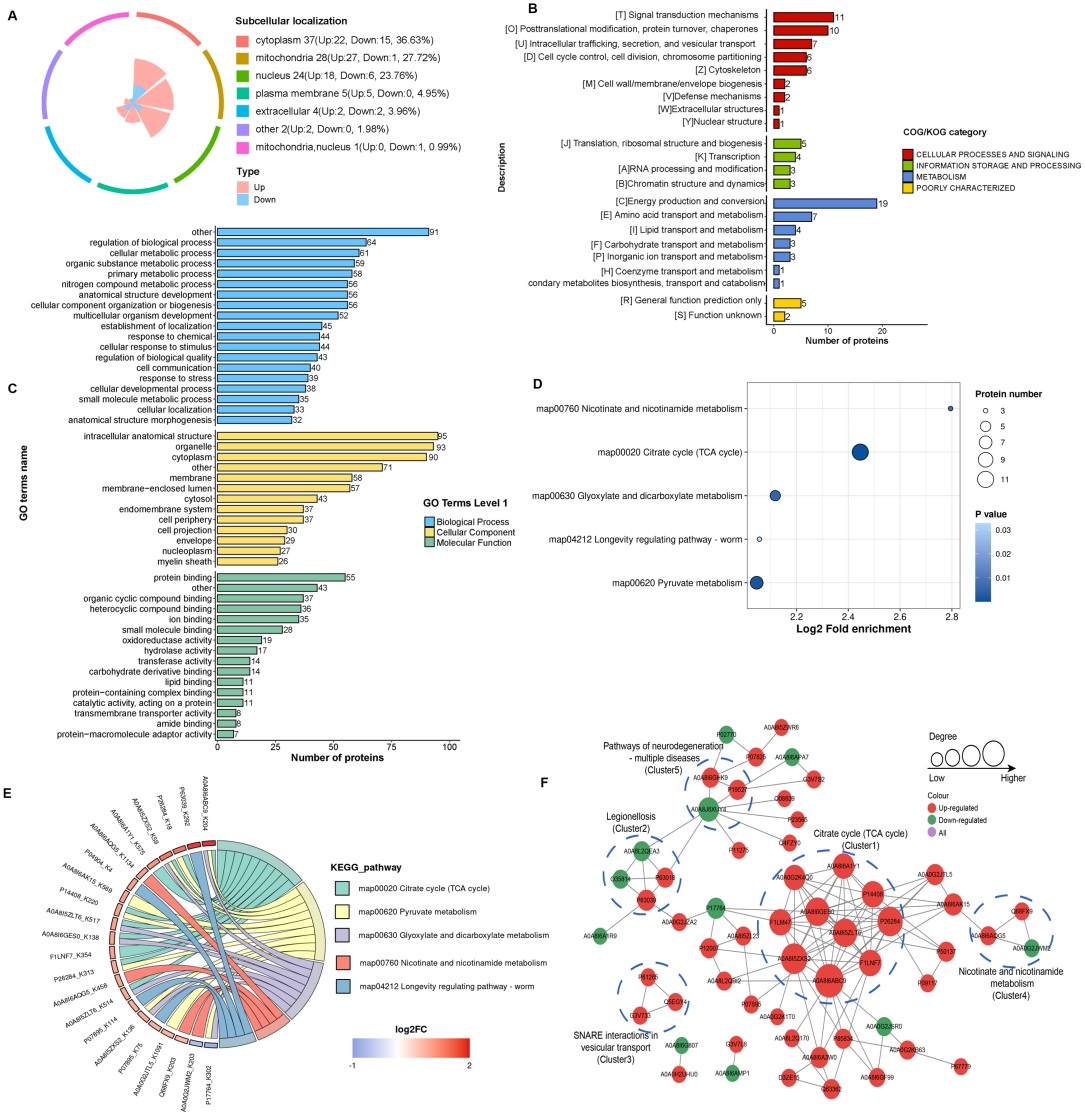
Functional classification and enrichment of lactylation modified proteins in the 4-VO and sham groups. **(A)** Subcellular localization of lactylation modified proteins. **(B)** Regulated-KOG classification. **(C)** Regulated-GO classification. **(D)** KEGG functional enrichment bubble chart. **(E)** KEGG functional enrichment chord diagram. 4-VO, four-vessel occlusion; KEGG, Kyoto Encyclopedia of Genes and Genomes; GO, Gene Ontology. **(F)** The PPI network of lactylated proteins, with red, green, and purple dots indicating proteins containing upregulated, downregulated, and both upregulated and downregulated sites, respectively. 4-VO, four-vessel occlusion; KEGG, Kyoto Encyclopedia of Genes and Genomes; GO, Gene Ontology; PPI, protein–protein interaction.

Subcellular localization analysis revealed that 36.6% of lactylated proteins in the 4-VO and Sham groups localized to the cytoplasm, 27.7% to the mitochondria, 23.8% to the nucleus, and 5.0% to the cell membrane (Figure 5A). Within the COG/KOG categories, 19, 11, 10, 7, and 7 lactylated proteins were enriched in energy production and conversion; signal transduction mechanisms; post-translational modifications; protein turnover, intracellular trafficking, secretion, and vesicular transport; and amino acid transport and metabolism, respectively (Figure 5B). Primary GO enrichment analysis of secondary lactylated proteins revealed that the biological process category exhibited enriched lactylated proteins, including 91, 64, 61, and 59 proteins in other, regulation of biological processes, cellular metabolic processes, and organic substance metabolic processes, respectively (Figure 5C). Subsequent KEGG pathway enrichment analysis of differentially expressed lactylated proteins revealed that these proteins were predominantly enriched in pathways including the citrate cycle (TCA cycle), pyruvate metabolism, and glyoxylate and dicarboxylate metabolism (Figures 5D,E). In the PPI network of the 4-VO group, 57 nodes were identified; 47 proteins were upregulated and 10 were downregulated (Figure 5F) compared with the sham group. Lactylated node proteins were primarily categorized as follows: legionellosis, SNARE interactions in vesicular transport, nicotinic acid and nicotinamide metabolism, and neurodegeneration pathways.

### 3.6 Functional enrichment of lactylation modified proteins in the 4-VO + EA and 4-VO groups

**Figure 6:**
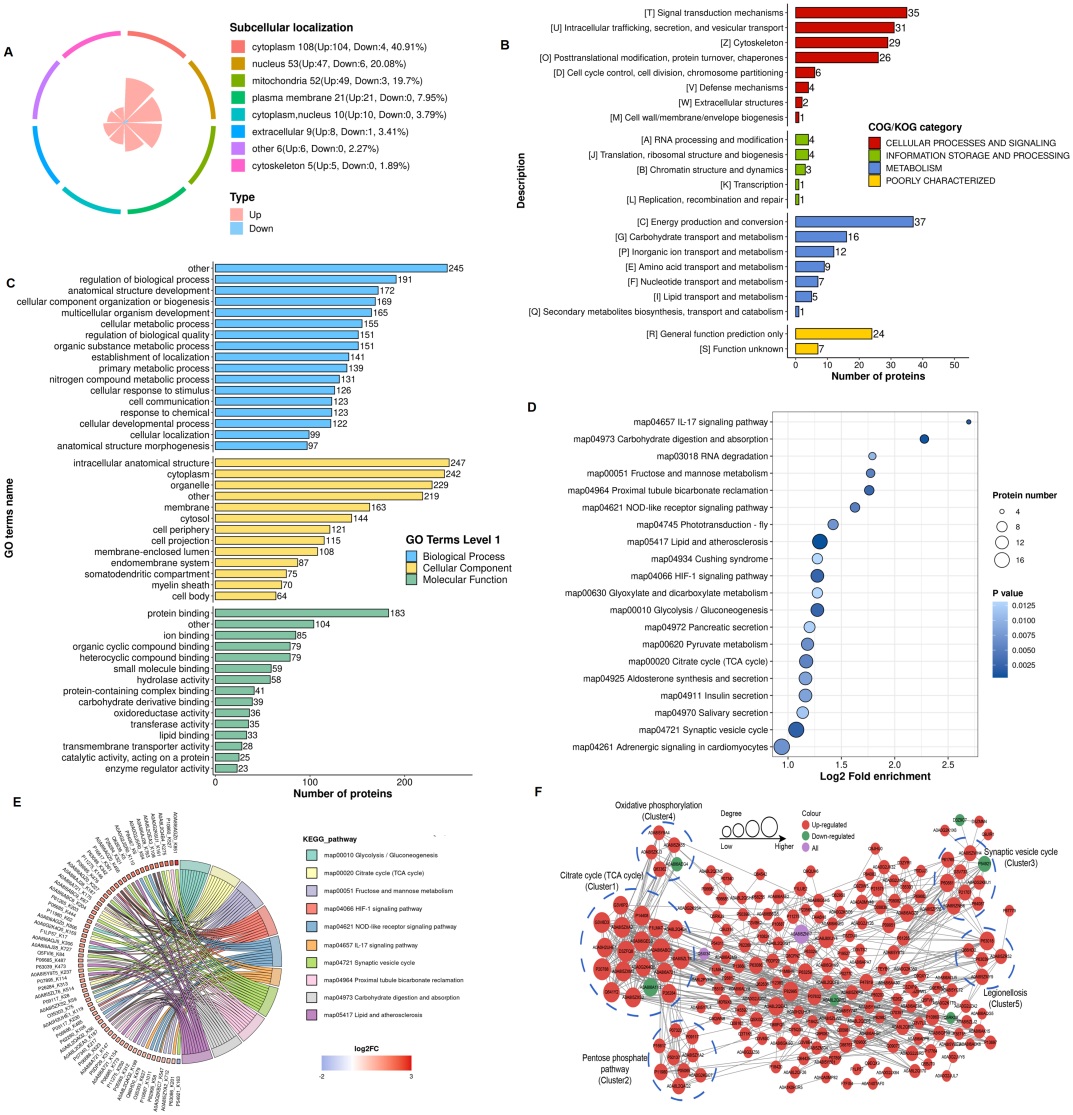
Functional classification and enrichment of lactylation modified proteins in the 4-VO + EA and 4-VO groups. **(A)** Subcellular localization of lactylation modified proteins. **(B)** Regulated-KOG classification. **(C)** Regulated-GO classification. **(D)** KEGG functional enrichment bubble chart. **(E)** KEGG functional enrichment chord diagram. 4-VO, four-vessel occlusion; EA, electroacupuncture; KEGG, Kyoto Encyclopedia of Genes and Genomes; GO, Gene Ontology. **(F)** The PPI network of lactylated proteins. 4-VO, four-vessel occlusion; KEGG, Kyoto Encyclopedia of Genes and Genomes; GO, Gene Ontology; PPI, protein–protein interaction.

Subcellular localization analysis revealed that the cytoplasm contained the most lactylated proteins (40.9%) in the 4-VO + EA and 4-VO groups, followed by the nucleus (20.1%), mitochondria (19.7%), and cell membranes (8.0%) (Figure 6A). The COG/KOG categories revealed the enrichment of 37, 35, 31, 29, and 26 lactylated proteins in energy production and conversion; signal transduction mechanisms; intracellular trafficking, secretion, and vesicular transport; the cytoskeleton; and post-translational modification, protein turnover, and chaperones, respectively (Figure 6B). The primary GO enrichment analysis of secondary lactylated proteins showed that in the biological process category, 245, 191, 172, and 169 lactylated proteins were enriched in other, regulation of the biological process, anatomical structure development, and cellular component organization or biogenesis, respectively (Figure 6C). Subsequently, KEGG pathway enrichment analysis of the differentially expressed lactylated proteins revealed that these proteins were mainly enriched in pathways such as lipid and atherosclerosis, hypoxia-inducible factor (HIF)-1 signaling, synaptic vesicle cycle, and NOD-like receptor signaling (Figures 6D,E). In the PPI network of the 4-VO + EA group, 205 nodes were identified; 197 proteins were upregulated, six were downregulated, and two contained upregulated and downregulated sites (Figure 6F) compared to the 4-VO group. Lactylated node proteins were primarily categorized as follows: citrate cycle (TCA cycle), pentose phosphate pathway, synaptic vesicle cycle, oxidative phosphorylation, and legionellosis. The functional enrichment analysis of lactylation-modified proteins in the 4-VO + EA and 4-VO groups is presented in Supplementary File 3.

### 3.7 Integrated lactylation proteomics profiling, KEGG pathway analysis, and Vdac3 verification in hippocampal tissue of VD rats following EA intervention

**Figure 7:**
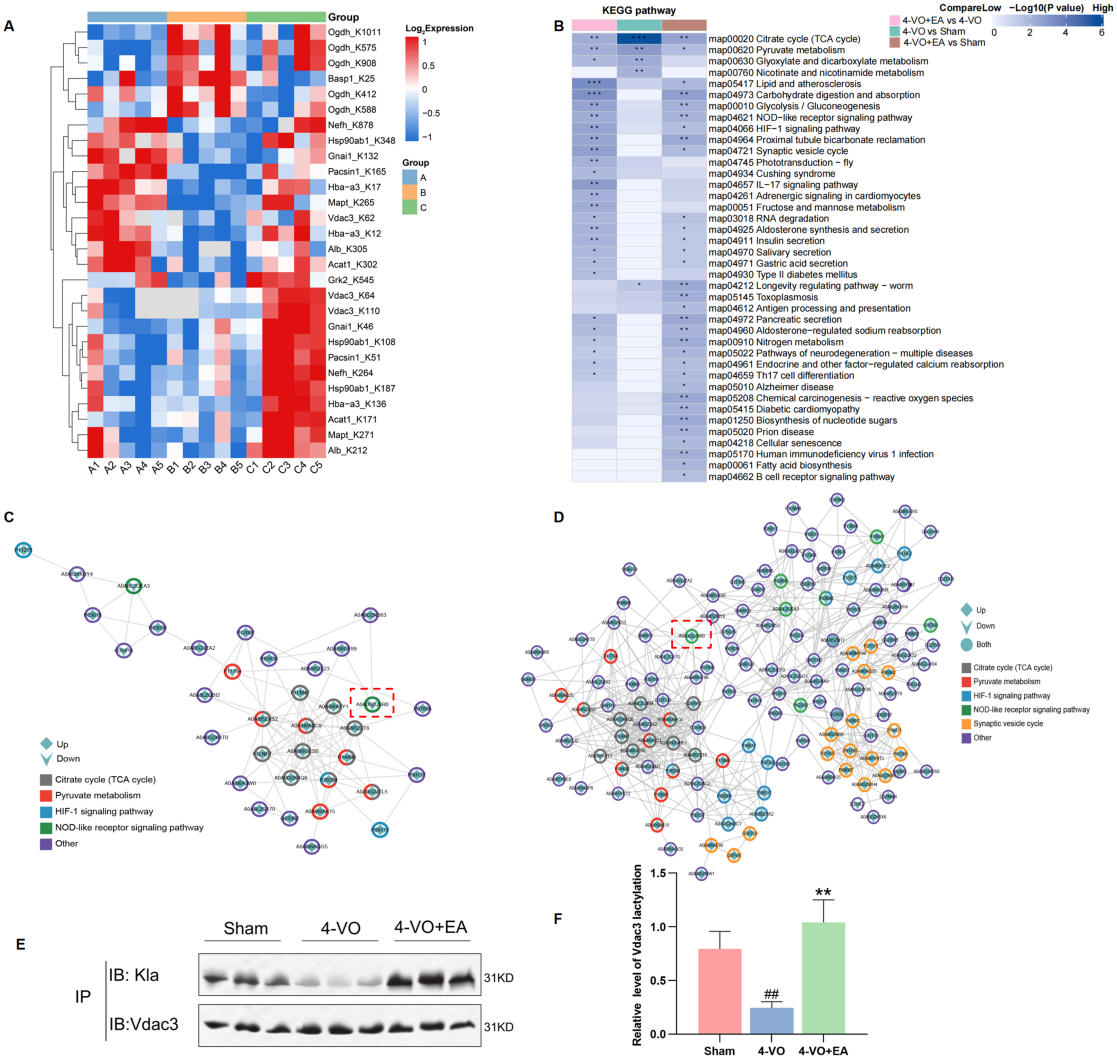
KEGG pathway clustering analysis of different comparison groups, the PPI network of differentially lactylated proteins in the pathways of interest, and verification of Vdac3 protein lactylation modifications. **(A)** Heatmap of lactylation sites for 12 key proteins. Each row represents a differentially modification site; each column represents a sample. Red indicates a high-level modification, blue indicates a low-level modification, and grey indicates sites that cannot be quantified in the corresponding samples. **(B)** The horizontal axis depicts different comparison groups, whereas the vertical axis indicates the enriched KEGG pathways. The colored blocks represent the functional descriptions of differentially expressed modified proteins enriched in each comparison group. Blue signifies high enrichment significance, whereas blue-white signifies low enrichment significance. ^*^p < 0.05, ^**^p < 0.01, and ^***^p < 0.001. **(C)** PPI network of differentially lactylated proteins involved in the 4-VO group and sham group in the five pathways of interest. **(D)** PPI network in the 4-VO group and 4-VO + EA group. The color of the outer circle indicates the corresponding KEGG pathway of the protein, whereas the shape within the circle denotes the upregulation or downregulation of the modification sites contained within the protein. A diamond shape signifies proteins with upregulated modification sites, a downward arrow indicates proteins with downregulated modification sites, and a circle signifies proteins with upregulated and downregulated modification sites. The red box indicates the Vdac3 protein. **(E)** The IP experiment verified the lactylation modification level of Vdac3. **(F)** Quantification was performed using ImageJ software, and the values are expressed as the mean with the corresponding standard error from three replicate experiments (*n* = 3, compared with the sham group, ^##^*p* < 0.01; compared with the 4-VO group, ^**^p < 0.01). 4-VO, four-vessel occlusion; EA, electroacupuncture; KEGG, Kyoto Encyclopedia of Genes and Genomes; PPI, protein–protein interaction; IP, immunoprecipitation; Vdac3, mitochondrial voltage-dependent anion channel protein3.

The lactylation proteomics study revealed that after electroacupuncture intervention, the lactylation sites of 12 differentially expressed proteins showed a gradually recovering trend, including modification sites on proteins such as on A0A0A0MP82 (Hba-a3), A0A0G2JSR0 (Vdac3), and A0A8I6A1Y1 (Ogdh). The complete protein designations and the specific lactylation sites showing gradually recovering trend are detailed in Figure 7A and Table 1. We propose that these lactylated proteins exhibiting gradually recovering trend may represent key therapeutic targets through which EA stimulation exerts its beneficial effects in VD. Using hierarchical clustering based on the Fisher’s exact test *p*-values obtained from the enrichment analysis, a heatmap was constructed to depict the associated functional groups in various comparison groups. The KEGG pathway cluster analysis of differentially expressed proteins in various comparison groups is shown in Figure 7B. The citrate cycle (TCA cycle) and pyruvate metabolism were observed in the 4-VO and sham groups, including the HIF-1 signaling pathway, synaptic vesicle cycle, and NOD-like receptor signaling pathway in the 4-VO + EA and 4-VO groups. Of particular significance, we constructed protein–protein interaction (PPI) networks for differentially lactylated proteins associated with significantly enriched pathways (Figures 7C,D) to probe deeper into their interaction patterns and identify potential key proteins. Crucially, Vdac3 (A0A0G2JSR0), a key mediator in the NOD-like receptor signaling pathway, was identified as a prominent hub node within these networks. Relative to the sham group, the lactylation level of Vdac3 in the 4-VO group was significantly decreased. Following EA intervention (4-VO + EA group), these modification levels were significantly higher compared to the 4-VO group.

**Table 1:** Protein identities, sites, and regulatory patterns of 12 key proteins related to lactylation.

Gene name	Protein accession	4-VO/Sham				4-VO + EA/4-VO			
		Position (Kla)	Ratio	*p*-value	Regulated type	Position (Kla)	Ratio	*p*-value	Regulated type
Hba-a3	A0A0A0MP82	K12 K17	0.6458 0.5083	0.0035 0.0015	Down	K136	2.8843	0.0272	Up
Vdac3	A0A0G2JSR0	K62	0.6341	0.0290	Down	K64 K110	2.8141 2.7650	0.0108 0.0267	Up
Ogdh	A0A8I6A1Y1	K575 K588 K908 K1011	1.8120 1.5412 1.5090 1.5355	0.0162 0.0103 0.0334 0.0045	Up	K412 K588	0.5719 0.6478	0.0346 0.0457	Up
Basp1	A0A8I6A304	K25	1.9609	0.0148	Up	K25	0.4665	0.0024	Up
Nefh	A0A8I6APA7	K878	0.4523	0.0105	Down	K264 K994	2.0411 2.0397	0.0200 0.0277	Up
Mapt	A0A8J8XUY4	K265	0.4133	0.0002	Down	K265 K271	1.8312 3.7812	0.0137 0.0046	Up
Hsp90ab1	A0A8L2QEA3	K348	0.5176	0.0027	Down	K108 K187	2.8668 1.9934	0.0105 0.0314	Up
Alb	P02770	K305	0.6441	0.0118	Down	K212	1.6401	0.0012	Up
Gnai1	P10824	K132	0.4313	0.0039	Down	K46	5.6040	0.0179	Up
Acat1	P17764	K302	0.5420	0.0461	Down	K171	2.3075	0.0470	Up
Grk2	P26817	K545	0.4688	0.0276	Down	K545	3.0196	0.0008	Up
Pacsin1	Q9Z0W5	K165	0.6443	0.0005	Down	K51	2.2043	0.0351	Up

Subcellular localization analysis revealed that differentially lactylation-modified proteins, including the mitochondrial protein Vdac3, predominantly located in the cytoplasm and mitochondria. As a predominant isoform of voltage-dependent anion channel (Vdac) protein in the mitochondrial outer membrane, Vdac3 functions as an essential regulator of mitochondrial physiology. Previous studies have confirmed (37, 38) that Vdac (particularly Vdac3) can regulate the inflammasome assembly and activation of NLRP3. Inhibiting the oligomerization of Vdac will disrupt the physical binding between Vdac and NLRP3, consequently reducing inflammatory cytokine release. Concurrently, there is evidence suggesting that aberrant NLRP3 inflammasome activation directly mediates neuronal and hippocampal cell pyroptosis, contributing significantly to vascular dementia pathogenesis (39, 40). Given the compelling association of differentially lactylated proteins with the NOD-like receptor signaling pathway, as clearly indicated by KEGG enrichment and clustering analyses (Tables 2, 3), coupled with Vdac3’s established role as a pivotal hub within this pathway, we prioritized it as the primary validation target for subsequent experimental investigation.

**Table 2:** Lactation modification of key proteins in the NOD-like receptor signaling pathway in the 4VO + EA and the 4VO group.

Gene name	Protein accession	Position (Kla)	4VO + EA/4VO ratio	Regulated type	Modified sequence
Vdac3	A0A0G2JSR0	K64	2.8141	Up	_YK[Lac (K)]VC[Carbamidomethyl (C)]NYGLIFTQK_
Vdac3	A0A0G2JSR0	K110	2.7650	Up	_LTVDTIFVPNTGK[Lac (K)]K_
Hsp90ab1	A0A8L2QEA3	K108	2.8668	Up	_ADLINNLGTIAK[Lac (K)]SGTK_
Hsp90ab1	A0A8L2QEA3	K187	1.9933	Up	_VILHLK[Lac (K)]EDQTEYLEER_
Dnm1l	O35303	K75	2.0853	Up	_RPLILQLVHVSPEDK[Lac (K)]R_
Dnm1l	O35303	K627	1.7006	Up	_SK[Lac (K)]PIPIMPASPQK_
Plcb1	P10687	K1011	1.6700	Up	_DK[Lac (K)]QQQQLLNLR_
Ywhae	P62260	K12	1.5261	Up	_EDLVYQAK[Lac (K)]LAEQAER_
Ywhae	P62260	K106	2.0277	Up	_LIC[Carbamidomethyl (C)]C[Carbamidomethyl (C)]DILDVLDK[Lac (K)]HLIPAANTGESK_
Ywhae	P62260	K118	1.6013	Up	_HLIPAANTGESK[Lac (K)]VFYYK_
Mapk1	P63086	K201	1.5435	Up	_APEIMLNSK[Lac (K)]GYTK_
Mapk1	P63086	K342	2.5827	Up	_LK[Lac (K)]ELIFEETAR_
Hsp90aa1	P82995	K58	1.6539	Up	_ELISNSSDALDK[Lac (K)]IR_

**Table 3:** Lactation modification of key proteins in the NOD-like receptor signaling pathway in the 4VO and the Sham group.

Gene name	Protein accession	Position (Kla)	4VO/Sham ratio	Regulated type	Modified sequence
Vdac3	A0A0G2JSR0	K62	0.6341	Down	_ASGNLETK[Lac (K)]YK_
Hsp90ab1	A0A8L2QEA3	K348	0.5176	Down	_APFDLFENK[Lac (K)]K_

To directly verify alterations in Vdac3 lactylation levels, we conducted co-immunoprecipitation (Co-IP) assays paired with high-specificity anti-lysine lactylation (Kla) antibodies (Figures 7E,F). Our results demonstrated a significant decrease in the level of Vdac3 lactylation modification in the 4-VO group compared to the sham group (*p* < 0.01), whereas EA intervention significantly increased Vdac3 lactylation levels (*p* < 0.01). Detailed statistics and the original WB experiment pictures can be found in Supplementary File 4. These experimental results aligned with prior 4D-Fast DIA lactylation quantitive data, jointly demonstrating that Vdac3 lactylation likely functions as a pivotal regulator modulating both NOD-like receptor signaling pathway and neuroprotection through electroacupuncture intervention in VD rats.

### 3.8 Analysis of lactylation site motifs in different groups and representative mass spectra of key proteins

**Figure 8:**
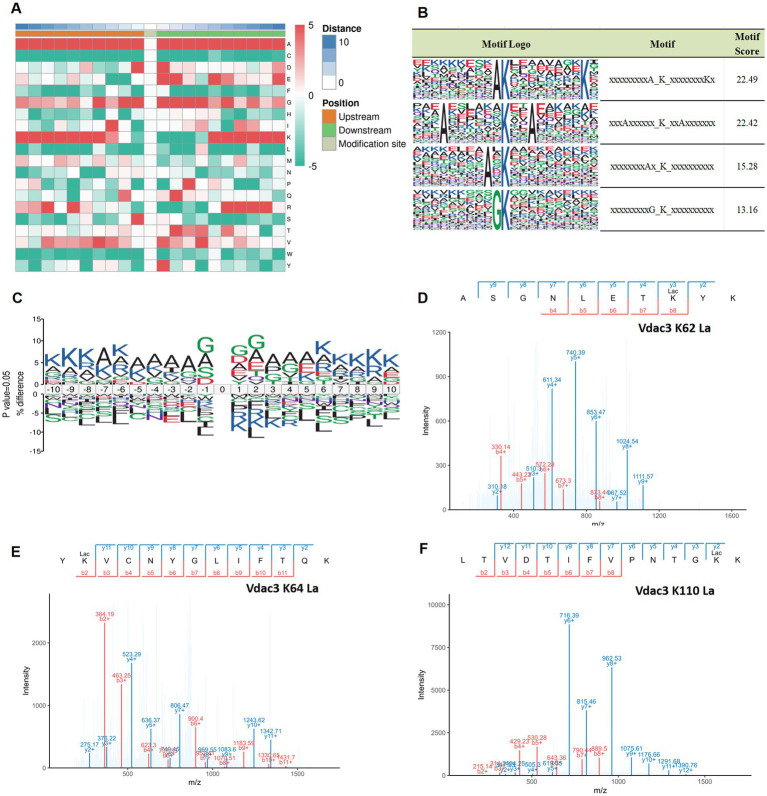
Analysis of lactylation site motifs in different groups and representative mass spectra of key proteins. **(A)** A heatmap of amino acids adjacent to lysine lactylation sites, with a green/red scale (−5 to 5) indicating the frequency of amino acid detection; red indicates high frequency; green indicates low frequency. The height ratio of amino acid abbreviation letters at specific positions reflects motif characteristics, with a % difference greater than 0 indicating a higher frequency of that amino acid at that position compared to the background, and less than 0 indicating a lower frequency. **(B)** Four conserved motif sites of lysine. **(C)** Sequence motif map of lactylation modification, 10 amino acids upstream and downstream of the modification site were selected, and the vertical axis indicates the difference between the proportion of the various amino acids at the same site in the experimental data set and the reference background, that is, percentage difference (%difference). The height ratio of amino acid abbreviation letters at specific positions represents the motif characteristics, and a %difference greater than 0 indicates that the frequency of the amino acid at this position is higher than the background. Values lower than 0 indicate that the frequency of the amino acid at this position is lower than the background. **(D–F)** The lactylation site of Vdac3 was identified by mass spectrometry. PPI, protein–protein interaction; 4-VO, four-vessel occlusion; EA, electroacupuncture.

To investigate the sequence preference for lactylation in hippocampal tissue, motif analysis was conducted on the 10 amino acids flanking lactylated lysine residues (−10 to +10) using the MoMo tool, which employs the motif-x algorithm. The analysis delineated the sequence characteristics of the modification site, as depicted in Figure 8A, which illustrates the amino acid distribution. High frequencies are indicated in red, whereas low frequencies are indicated in green. Modification site motif analysis revealed specific amino acid preferences flanking lactylation sites. Furthermore, sequence analysis identified four predominant motifs surrounding lactylated lysine residues: xxxxxxxxA_K_xxxxxxxxKx, xxxAxxxxxx_K_xxAxxxxxxx, xxxxxxxAx_K_xxxxxxxxxx, and xxxxxxxxxG_K_xxxxxxxxxx. These patterns demonstrate the conservation of lactylation motifs in hippocampal tissue, with preferential positioning of alanine (A), lysine (K), and glycine (G) residues relative to the modified lysine, as illustrated in Figure 8B. These findings indicate that lactylation modification exhibits significant sequence conservation within hippocampal tissue, characterized by the presence of a core phenomenon of A/G enrichment near the modification sites, which may affect the recognition of the enzyme or the conformation of the protein. Furthermore, motif visualization (Figure 8C) strongly supports these observations. By calculating site-specific amino acid frequency differences relative to background sequences and representing these differences with letter sizes, it is clearly visible that the content of G and A is significantly higher than the background level at multiple positions surrounding the lactylation sites.

The enrichment of A/G may reflect binding preference of lactoyl-modifying enzymes for substrates, similar to the way acetyltransferases recognize the “GK” sequence (41). Alanine (A), notably present in conventional secondary structures like β-sheets, may influence the local microenvironment due to its hydrophobicity (42). The side chain of glycine (G) is extremely short, which may enhance the conformational flexibility of the peptide chain backbone (43). The enrichment of them at the modification sites may reflect that the lactylation effect tends to favor specific secondary structural regions or flexible areas of the protein. This suggests that potential lactate transferases, such as p300/CBP, which has demonstrated lactylation catalytic activity (44), may possess specific recognition features for the substrate sequence. The enrichment of lysine (K) itself may suggest that adjacent lysines (such as potential acetylation sites) have regulatory interactions with the lactylation sites (45, 46). However, these hypotheses regarding enzyme-substrate recognition and regulation still need to be confirmed through further research.

It is worth noting that among the 12 key differentially lactylated proteins identified earlier, we focused on alterations at lactylated lysine residues of Vdac3 (A0A0G2JSR0). Compared to the sham group, the lactylation level at lysine 62 (K62) of Vdac3 protein was reduced in the 4-VO group. However, after electrical stimulation intervention, the lactylation levels at lysine 64 (K64) and lysine 110 (K110) sites significantly increased. These site-specific changes represent EA-mediated, specific modulation of Vdac3 lactylation. The representative tandem mass spectrometry (MS/MS) profiles of these different lactylation sites are shown in Figures 8D–F.

## 4 Discussion

The severe and progressive cognitive impairment caused by VD poses a major challenge to human quality of life. As its global incidence rate rises and its impact on younger populations becomes increasingly prominent (47), finding effective treatment methods and identifying therapeutic targets are of crucial importance for the management of vascular dementia. Previous studies have determined that EA at the Sishencong (Ex-HN01) and bilateral Fengchi (GB 20) points showed favorable efficacy in treating VD during long-term clinical practice (28). The Sishencong acupuncture point (Ex-HN01) adjusts yin and yang, enhances intelligence, and calms the mind. It is situated in the projection area, which is closely associated with advanced cognitive functions, such as thinking, memory, and spirit, specifically within the forehead, temporal, and parietal lobes. At this point, acupuncture can enhance the blood supply to the anterior circulation of the brain. The Fengchi acupuncture point (GB 20) is capable of awakening the mind, opening the orifices, and enhancing vision and intelligence. Acupuncture at this point can promote blood supply to the vertebrobasilar artery system and the posterior circulation of the brain. Numerous studies (10, 12, 48, 49), have demonstrated that EA can mitigate neuroinflammation, regulate synaptic plasticity, alleviate neural damage, and preserve brain tissue. However, the pathological mechanisms by which EA ameliorates learning and memory impairments observed in the vascular dementia rat model remains to be fully elucidated.

Notably, the pathogenesis of VD is strongly associated with cerebral ischemia and hypoxia resulting from chronic cerebral hypoperfusion (50, 51), in which lactate metabolism also plays a key role. In the brain, lactate production occurs predominantly in astrocytes, where it is transported to neurons to fulfill the brain’s energy requirements (52). In addition to its role in energy provision, when intracellular lactate concentration reaches a certain level, it also causes the lysine residues in histone and non-histone proteins to undergo lactylation modification (53). Zhao et al. (20) first identified lysine lactylation modifications using liquid chromatography-mass spectrometry. These modifications in protein lactylation could potentially affect energy production and conversion along with intracellular trafficking, secretion, and vesicular transport (54, 55), thereby playing a role in the onset and progression of VD. Lactylation modifications have been confirmed in various cell types and play a pivotal role in gene expression regulation (56), inflammatory responses (57), construction of the tumor microenvironment (58), maintenance of cardiovascular function (59), and neural excitation (24). With the discovery of the involvement of lactate in various biological processes, its role in the central nervous system has garnered increasing attention (22). The accumulation of lactic acid promotes the lactylation modification on lysine residues (60). These modifications play critical roles in both physiological and pathological environments of brain tissues, influencing its energy metabolism and signal transduction (55). Furthermore, Yao et al. (61) recently verified the involvement of lactylation modifications in the pathological processes of cerebral ischemia-reperfusion injury, suggesting that Kla may be one of the critical regulatory mechanisms mediating the core pathological features of VD-cerebral ischemia and hypoxia. Given that Kla has clear biological significance and plays a specific role in the pathological processes related to cerebral ischemia/hypoxia, and no research has been conducted on the role of protein lactylation modifications in the mechanism of EA intervention in VD. Therefore, we first employed 4D-Fast DIA lactylation modification quantitative proteomics analysis to clarify the potential role of protein lysine lactylation in VD rats.

In the present study, the 4-VO method was used as a vascular dementia model, which confirmed that EA at the Sishencong (Ex-HN01) and Fengchi (GB 20) acupoints significantly ameliorated cognitive dysfunction in VD rats. Eight-week-old male Sprague–Dawley (SD) rats were selected to establish the model; given that estrogen can modulate cerebral blood flow via vasodilation and exert neuroprotective effects (62), male rats were chosen to avoid potential interference from endogenous hormones when studying the mechanism of cerebral ischemic injury. Secondly, this age group (8-week-old adult rats) can address the issue of immature nervous systems in young rats and reduce the risk of spontaneous degenerative changes in old rats, thereby ensuring the baseline stability of neural function. The 4-VO method represents an internationally recognized and is maturely used to induce VD models (63, 64). It can effectively provide a 24-h low perfusion adaptation period, combined with a clear pre-occlusive stage of cerebral ischemia and the subsequent reperfusion stage. Repeated ischemia-reperfusion cycles and continuous chronic hypoperfusion are the pathogenic factors of VD, and the 4-VO model has a higher correlation with the clinical VD pathogenesis (65). However, the survival rate of this method is relatively low. Previous research (66) indicates that the two-vessel occlusion (2-VO) method achieves lower mortality than 4-VO, while 4-VO induces significantly more pronounced ischemic alterations in hippocampal neurons. Since the 4-VO method provides a superior modeling platform for replicating core features of VD in rats, it was selected for the present investigation. Our research group has long employed established electroacupuncture stimulation parameters comprising a continuous wave at 2 Hz frequency and 1 mA intensity (67). This 2-Hz stimulation is classified as low-frequency electro-acupuncture stimulation, which can effectively activate the endogenous opioid system (such as enkephalin), promoting neuroprotective effects and reducing neuroinflammatory responses (68). The pain perception threshold in rats typically ranges between 1–2 mA. The selection of a stimulation intensity of 1 mA was to ensure effective activation of deep nerve fibers at the acupoints while avoiding stimulation of nociceptive C fibers (69), thereby reducing interfering factors. In the Morris water maze (MWM) testing, the swimming speeds of all experimental groups were systematically quantified. Statistical analysis revealed no significant intergroup differences in mean swimming speed, which confirmed that non-cognitive variables (such as motor function) do not affect the effectiveness of spatial learning and memory assessment. Therefore, the cognitive function indicators derived from the maze memory task have extremely high specificity and reliability. Behavioral assessments demonstrated that electroacupuncture significantly ameliorated spatial learning and memory deficits in VD rats. Histopathological examination via HE staining revealed that EA intervention partially restored hippocampal cytoarchitecture in VD rats. The ELISA quantitative analysis further confirmed that the levels of inflammatory mediators in the hippocampal tissues of VD rats that received EA intervention were reduced. Additionally, WB analysis using a pan-lactylation antibody detected moderate alterations in lysine lactylation levels within hippocampal tissues of the 4-VO + EA group; these alterations may be related to the regulatory function of the Kla-mediated inflammatory pathway (70).

When combined with the 4D-Fast DIA-based quantitative lactyl-proteomics, the EA intervention significantly altered the modification profiles of Kla in the hippocampus, identifying 8,852 Kla sites across 2,786 proteins. The differentially modified Kla sites were predominantly enriched within three core signaling pathways. (1) Energy metabolism pathways (citrate cycle/pyruvate metabolism). Given that lactate is the end product of glycolysis, it may serve as a crucial energy substrate in the brain (71). Intracellular lactate production occurs primarily through glycolysis and glutamine metabolism (72). Lactate can be metabolized to pyruvate, which enters the TCA cycle or is converted to lactyl-CoA, a substrate for protein lactylation. The precise mechanisms by which protein lactylation modulates the TCA cycle and pyruvate metabolism and subsequently contributes to VD pathogenesis warrant further investigation. (2) Synaptic plasticity pathways (synaptic vesicle cycle), where Kla may influence synaptic transmission by regulating neurotransmitter release mechanisms. Supporting this, Yan et al. (73) demonstrated that that emulsification of synapsomal protein 91 (SNAP91) improves synaptic structure formation and nerve activity in the medial prefrontal cortex. Wang et al. (74) demonstrated an interaction between synaptophysin and V-ATPase in synaptic vesicles, whereby synaptophysin regulates the V-ATPase abundance and consequently influences synaptic vesicle biogenesis and function. The enrichment of intracellular trafficking, secretion, and vesicular transport pathways (COG) suggests a potential role of protein lactylation in the regulation of neurotransmitter storage and release within synaptic vesicles. (3) Hypoxia-inflammation axis (HIF-1/NOD-like receptor pathway). The HIF-1 signaling pathway serves as a pivotal regulatory mechanism that is crucially involved in the physiological and pathological cellular responses to fluctuations in oxygen availability (75). HIF-1 directly regulates the expression of target genes involved in cellular energy metabolism and the maintenance of cellular homeostasis (76), and its dysregulation contributes to VD pathogenesis. In cerebral ischemic diseases, the NOD-like receptor thermal protein domain associated protein 3 (NLRP3) inflammasome constitutes a critical downstream target of hypoxia-inducible factor (HIF)-1α (77). NOD-like receptors (NLRs) constitute the largest family of cytosolic pattern recognition receptors. Upon the recognition of pathogen-associated molecular patterns or damage-associated molecular patterns, NLRs self-oligomerize to form large signaling complexes, including NLRP3 and NLRC4 inflammasomes, and the NOD2 nodosome. This subsequently activates NF-κB and MAPK signaling pathways, and induces pyroptosis, resulting in the release of inflammatory cytokines and triggering downstream immune and inflammatory responses (78). Su et al. (79) proposed that high lactylation levels of H3K18 effectively induce upregulation of ALKBH5. This enzyme reduces m6A modification on NLRP3 mRNA, destabilizing its transcript and thereby suppressing inflammasome activation. Although the involvement of HIF-1 (80), synaptic vesicle cycling (81) and NOD-like receptor signaling (82) in VD pathogenesis has been established, the precise roles of specific protein lactylation modifications require further elucidation.

We prioritized 12 differentially expressed proteins with significant reversal of lactylation modification sites following EA intervention, including Hba-a3, Vdac3, Ogdh, and Pacsin1. Critical observations focused on Pacsin1 within synaptic vesicle cycle pathways. Protein kinase C and casein kinase substrate in neurons protein 1 (Pacsin1) is a key regulator of synaptic vesicle cycling (83). In this study, Pacsin1, exhibited a trend of recovering its lactylation modification after electroacupuncture intervention. It plays a crucial role in the synaptic vesicle cycle/endocytosis and microtubule dynamic reorganization to maintain axonal plasticity (84). The alteration in the lactylation state is likely to represent the key molecular mechanism by which Pacsin1 regulates these processes to promote neuronal signal transmission; however, the specific mechanisms involved still need to be further verified. Furthermore, we highlight Vdac3, a key protein involved in the NOD-like receptor signaling pathway, identified as a critical hub in the PPI network. Mitochondrial Vdac is an important channel protein on the outer mitochondrial membrane. Vdac3 plays a crucial role in cellular bioenergetics. Its dysregulation can significantly reduce TCA cycle intermediates (85), leading to mitochondrial dysfunction and subsequent NLRP3 inflammasome activation. Furthermore, Vdac has a strong permeability to Ca^2+^, and the increased expression of Vdac leads to calcium ion overload in mitochondria, which can cause mitochondrial damage. The elevated matrix calcium drives the oligomerization of Vdac on the outer membrane, and Vdac forms macromolecular-sized pores in the outer mitochondrial membrane, allowing proteins and mtDNA to leave the mitochondria and recruit NLRP3 to the Vdac-NLRP3 complex on the mitochondrial surface, which is usually associated with apoptosis and inflammation (86). Studies have demonstrated the involvement of voltage-dependent anion channels (Vdacs) in NLRP3 inflammasome assembly and activation (37, 38).

The pivotal discovery in this investigation resides in our direct verification, through co-immunoprecipitation (IP) and western blot (WB) techniques, that the lactylation modification levels of VDAC3 protein exhibit significant alterations across experimental groups. This result demonstrates remarkable consistency with the lactyl-modification profiles obtained from 4D-Fast DIA quantitative proteomic analyses. Therefore, we propose the following hypothesis: the alterations in lactylation levels at specific Vdac3 sites likely constitute a key therapeutic target for ameliorating cognitive dysfunction in VD. Specifically, electroacupuncture intervention will enhance the lactylation effect at different lysine residues of Vdac3, thereby affecting its oligomerization state. This regulatory effect subsequently regulates the NOD-like receptor signaling pathway and inhibits its excessive activation. Collectively, this cascade may represent one of the key molecular mechanisms underlying electroacupuncture-induced improvement of cognitive dysfunction in VD rats. The precise regulatory mechanisms of Vdac3 lactylation and its role in neuroinflammation warrant further in-depth investigation in future studies.

The pattern analysis in this study revealed a significant sequence preference around the lysine emulsification sites, particularly in the adjacent regions, which are rich in alanine (A) and glycine (G). This finding suggests that the substrate specificity of lactylation may not only be regulated by the catalytic action of the enzyme, but could also be influenced by the sequence context (87). The presence of small amino acids such as A and G may contribute to the flexibility of the protein, thereby enabling the binding and catalytic modification of lactoyl transferase enzymes. Further analysis revealed that after the intervention of EA, the enrichment level of the relevant motifs increased, suggesting that EA may regulate the lactylation process in specific sequence environments, thereby influencing the functional state of the target protein. Previous studies (88, 89) have demonstrated that motif characteristics surrounding lysine modification sites are closely linked to protein function. The presence of specific motifs can determine a protein’s role in critical pathways such as energy metabolism and signal transduction. Thus, our motif analysis not only aids in predicting novel lactylation substrates but also lays the foundation for further exploration of the molecular mechanism by which EA regulates lactylation modification and the broader functional regulatory network involved.

Although this study utilized 4D-Fast DIA quantitative proteomics to investigate the significance and associated mechanisms of lactylation, there are still some significant limitations. First, the assessment of apoptotic and inflammatory alterations in hippocampal tissue was limited to initial morphological observations based on H&E staining. Quantitative analyses employing specific markers for apoptosis were not performed. This methodological gap potentially compromises the precision of our understanding of the underlying cellular mechanisms. Therefore, subsequent studies will use specific quantitative methods for verification. Second, regarding quality control, data verification procedures will be further refined. For specific data points, such as Sample A1, more stringent quality control will be implemented prior to downstream analysis to ensure the robustness and reproducibility of findings. Third, this study only employed 8-week-old male rats as the experimental model. While this approach helps reduce confounding effects from endogenous variables such as hormonal fluctuations and enhances data stability and comparability, it nonetheless introduces certain limitations in translating the findings to broader populations. Given the common clinical occurrence of VD in the elderly population and its similar prevalence in both sexes, conclusions drawn from single-sex and single-age animal models may introduce inherent biases when generalized to different populations. Future research should incorporate both male and female rats and varied age cohorts in rat models to enhance the generalizability and clinical translatability of results. Finally, the limited sample size may reduce statistical power, and the absence of a dedicated EA intervention group in normal healthy rats precludes to draw clear conclusions on the pathophysiological specificity of the electrical stimulation effect. Future studies should expand the sample size, replicate our experiments verify them under different experimental conditions, and clarify the potential roles of other biomarkers and signaling pathways to gain a deeper understanding of the role of protein emulsification in the pathogenesis of vascular dementia and the therapeutic mechanism of electrical stimulation intervention.

## 5 Conclusion

The distribution and abundance of protein lactylation modification sites in the hippocampal tissues of the sham, 4-VO, and 4-VO + EA groups were comprehensively analyzed using whole-proteome lactylation sequencing. The main biological pathways regulated by Kla were identified, including the citrate cycle (TCA cycle), NOD-like receptor signaling pathway, and synaptic vesicle cycle. The primary objective of this study was to identify the major lactylation-modified proteins (Vdac3 and Pacsin1) involved in VD intervention through EA. This included determining their relationship with the occurrence of VD and the mechanism of EA intervention, with the aim of identifying potential clinical targets. In summary, our research provides new insights into the physiological functions of Kla and the mechanisms of EA intervention in VD. However, further research is necessary to confirm the specific mechanisms by which lactylation modifications influence the progression of VD.

## Data Availability

The original contributions presented in the study are included in the article/Supplementary material, further inquiries can be directed to the corresponding author.

## Footnotes

^1^ https://www.ebi.ac.uk/interpro/entry/pfam/#table

^2^ http://www.genome.jp/kegg/kaas/
