# Supplementary material for: Effect of electroacupuncture on hippocampal protein lactylation in a rat model of vascular dementia
Source: Front Neurol. 2025 Sep 2;16:1629474. doi: 10.3389/fneur.2025.1629474 (PMC12439496; doi:10.3389/fneur.2025.1629474)
Supplement: Supplementary file 1 [file Data_Sheet_1.docx]

**Table 1. Comparison of Swimming Speed Across Experimental Groups**

（$\bar{x}\pm s$, *n*=8, cm/s）

| Group | Pre-intervention | Post-intervention |
| --- | --- | --- |
| Sham | 16.62±3.17 | 16.40±2.71 |
| 4-VO | 15.89±2.57 | 16.16±3.17 |
| 4-VO+EA | 16.67±2.90 | 16.21±3.85 |
| *F* | 0.185 | 0.012 |
| *P* | 0.832 | 0.988 |
| *P*1-value | 0.616 | 0.886 |
| *P*2-value | 0.973 | 0.909 |
| *P*3-value | 0.592 | 0.977 |

**Note:**Data are expressed as mean ± standard deviation. All variables satisfied the assumptions of normality (Shapiro-Wilk test, *P* > 0.05) and homogeneity of variance (Levene's test, *P* > 0.05).*P*1-values denote comparisons between Sham vs. 4-VO groups;*P*2-values denote comparisons between Sham vs. 4-VO+EA groups;*P*3-values denote comparisons between 4-VO vs. 4-VO+EA groups.

**Table 2. Pre-intervention Escape Latency Across Groups**

（$\bar{x}\pm s$, *n*=8, s）

| Group | D1 | D2 | D3 | D4 | D5 |
| --- | --- | --- | --- | --- | --- |
| Sham | 50.64±8.72 | 38.04±7.97 | 29.12±6.05 | 25.43±5.45 | 22.22±3.83 |
| 4-VO | 67.66±9.71^##^ | 60.73±3.23^##^ | 57.43±5.25^##^ | 55.64±7.93^##^ | 53.14±5.90^##^ |
| 4-VO+EA | 66.79±9.83^##^ | 61.57±7.33^##^ | 59.87±4.19^##^ | 56.02±4.35^##^ | 54.65±5.10^##^ |
| *P*1-value | 0.005 | ＜0.001 | ＜0.001 | ＜0.001 | ＜0.001 |
| *P*2-value | 0.008 | ＜0.001 | ＜0.001 | ＜0.001 | ＜0.001 |
| *P*3-value | 1.000 | 1.000 | 1.000 | 1.000 | 1.000 |

**Note:** All datasets satisfied normality (Shapiro-Wilk, *P* > 0.05) and homogeneity of variance (Levene’s, *P* > 0.05) assumptions. Repeated-measures ANOVA with Mauchly’s sphericity test (χ2= 7.862, *P* = 0.377) confirmed sphericity (no correction applied). Significant effects were observed: group (*F* = 144.117, *P* < 0.001), time (*F* = 32.253, *P* < 0.001), and group * time interaction (*F* = 2.805, *P*= 0.008). Simple effects analysis with LSD tests was employed to assess between-group differences at identical time points and within-group changes across distinct time nodes. *P*1 (Sham vs. 4-VO), *P*2 (Sham vs. 4-VO+EA), *P*3 (4-VO vs. 4-VO+EA); ^##^*P* < 0.01 versus Sham group.

**Table 3. Post-intervention Escape Latency Across Groups**

（$\bar{x}\pm s$, *n*=8, s）

| Group | D1 | D2 | D3 | D4 | D5 |
| --- | --- | --- | --- | --- | --- |
| Sham | 38.54±5.40 | 28.72±4.39 | 25.27±2.31 | 21.51±3.57 | 21.04±4.05 |
| 4-VO | 61.03±7.51^##^ | 54.45±9.18^##^ | 45.62±3.78^##^ | 43.35±4.14^##^ | 43.88±6.81^##^ |
| 4-VO+EA | 54.73±7.53^##^ | 43.78±4.27^##**^ | 35.01±6.18^##**^ | 25.64±2.27^##**^ | 23.65±4.65^##**^ |
| *P*1-value | ＜0.001 | ＜0.001 | ＜0.001 | ＜0.001 | ＜0.001 |
| *P*2-value | ＜0.001 | ＜0.001 | ＜0.001 | 0.075 | 1.000 |
| *P*3-value | 0.245 | 0.009 | ＜0.001 | ＜0.001 | ＜0.001 |

**Note:** All datasets satisfied normality (Shapiro-Wilk, *P* > 0.05) and homogeneity of variance (Levene’s, *P* > 0.05) assumptions. Repeated-measures ANOVA with Mauchly’s sphericity test (χ2= 12.286, *P* = 0.199) confirmed sphericity (no correction applied). Significant effects were observed: group (*F* = 60.926, *P* < 0.001), time (*F* = 383.066, *P* < 0.001), and group * time interaction (*F* = 2.972, *P*= 0.006). Simple effects analysis with LSD tests was employed to assess between-group differences at identical time points and within-group changes across distinct time nodes. *P*1 (Sham vs. 4-VO), *P*2 (Sham vs. 4-VO+EA), *P*3 (4-VO vs. 4-VO+EA); ^##^*P* < 0.01 versus Sham group.^**^*P* < 0.01 versus 4-VO group.

**Table 4. Platform Crossings Across Groups**

（$\bar{x}\pm s$, *n*=8, times）

| Group | Pre-intervention | Post-intervention |
| --- | --- | --- |
| Sham | 7.75±1.39 | 7.88±1.46 |
| 4-VO | 2.50±0.93^##^ | 2.75±1.28^##^ |
| 4-VO+EA | 2.25±1.03^##^ | 6.875±1.67^**^ |
| *P*1-value | ＜0.001 | ＜0.001 |
| *P*2-value | ＜0.001 | 0.429 |
| *P*3-value | 1.000 | ＜0.001 |

**Note:** All datasets satisfied normality (Shapiro-Wilk, *P* > 0.05) and homogeneity of variance (Levene's, *P* > 0.05) assumptions. Repeated-measures ANOVA with Mauchly's sphericity test (*P* < 0.05) indicated violation of sphericity, requiring Greenhouse-Geisser correction. After correction, significant effects were observed: group (*F* = 78.091, *P* < 0.001, partial η² = 0.881), time (*F* = 15.408, *P* < 0.001, partial η² = 0.423), and group × time interaction (*F* = 12.065, *P* < 0.001, partial η² = 0.535). Simple effects analysis with LSD tests was employed to assess between-group differences at identical time points and within-group changes across distinct time nodes. *P*1 (Sham vs. 4-VO), *P*2 (Sham vs. 4-VO+EA), *P*3 (4-VO vs. 4-VO+EA); ^##^*P* < 0.01 versus Sham group.^**^*P* < 0.01 versus 4-VO group.
