# Supplementary material for: Effect of electroacupuncture on hippocampal protein lactylation in a rat model of vascular dementia
Source: Front Neurol. 2025 Sep 2;16:1629474. doi: 10.3389/fneur.2025.1629474 (PMC12439496; doi:10.3389/fneur.2025.1629474)

**Table Comparison of Hippocampal TNF-α, IL-1β, and IL-18 Levels Across Experimental Groups**

（$\bar{x}\pm s$, *n*=5，pg/mL）

| Group | TNF‐α | IL-1β | IL-18 |
| --- | --- | --- | --- |
| Sham | 37.53±4.33 | 8.74±1.49 | 19.33±4.99 |
| 4-VO | 58.21±10.30^##^ | 20.06±1.67^##^ | 48.97±12.20^##^ |
| 4-VO+EA | 44.99±7.55^*^ | 9.64±1.94^**^ | 25.73±6.63^**^ |
| *F* | 9.052 | 67.654 | 16.781 |
| *P* | 0.004 | ＜0.001 | ＜0.001 |
| *P*1-value | 0.001 | ＜0.001 | ＜0.001 |
| *P*2-value | 0.156 | 0.42 | 0.258 |
| *P*3-value | 0.02 | ＜0.001 | 0.001 |

**Note:**Data are expressed as mean ± standard deviation. All variables satisfied the assumptions of normality (Shapiro-Wilk test, *P* > 0.05) and homogeneity of variance (Levene's test, *P* > 0.05). *P*1-values denote comparisons between Sham vs. 4-VO groups;*P*2-values denote comparisons between Sham vs. 4-VO+EA groups;*P*3-values denote comparisons between 4-VO vs. 4-VO+EA groups.^##^*P* < 0.01 versus Sham group.^*^*P*＜0.05, ^**^*P* < 0.01 versus 4-VO group.

**Lactylation modification pan-antibody original picture**

Coomassie brilliant blue staining


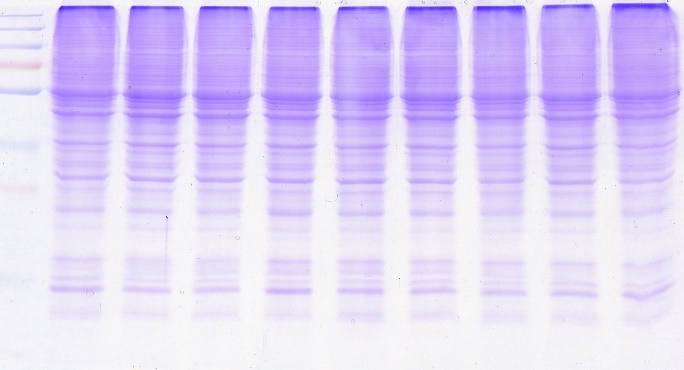


La-30


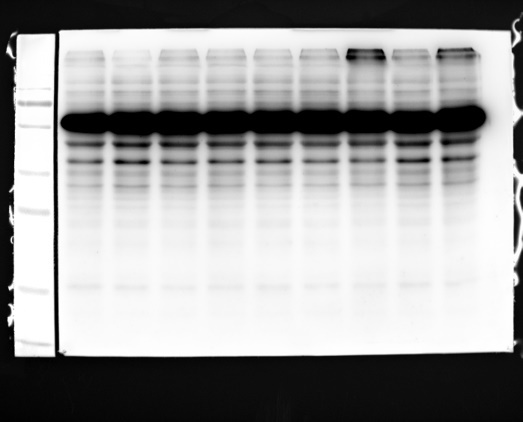


**La-60**


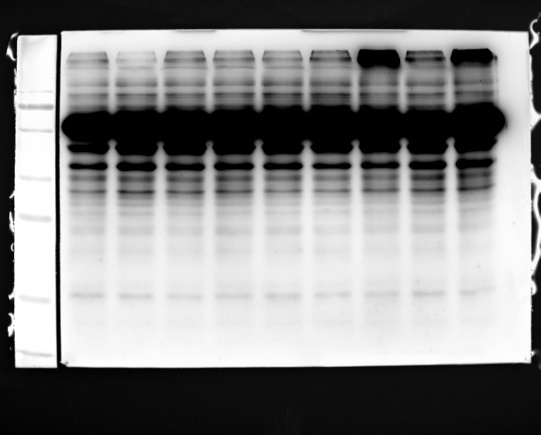

Supplement: Supplementary file 2 [file Data_Sheet_2.docx]
