# Supplementary material for: Effect of electroacupuncture on hippocampal protein lactylation in a rat model of vascular dementia
Source: Front Neurol. 2025 Sep 2;16:1629474. doi: 10.3389/fneur.2025.1629474 (PMC12439496; doi:10.3389/fneur.2025.1629474)
Supplement: Supplementary file 3 [file Data_Sheet_3.docx]

## **Functional Enrichment of Lactylation Modified Proteins in the 4-VO+EA and Sham Groups**

Subcellular localization analysis revealed that 39.8% and 23.4% of the lactylated proteins in the 4-VO+EA and 4-VO groups were localized in the cytoplasm and nucleus, respectively. Furthermore, 20.1% and 7.3% were found in mitochondria and cell membranes, respectively (Fig. S1A). In the COG/KOG categories, 66, 59, 54, 52, and 46 lactylated proteins were enriched in signal transduction mechanisms; energy production and conversion; general function prediction only; intracellular trafficking, secretion, and vesicular transport; and post-translational modification, protein turnover, and chaperones, respectively (Fig. S1B). The primary GO enrichment analysis of secondary lactylated proteins showed that in the biological process category, 443, 356, 288, and 283 lactylated proteins were enriched in other, regulation of the biological process, cellular component organization or biogenesis, and anatomical structure development, respectively (Fig. S1C). Subsequently, KEGG pathway enrichment analysis of differentially expressed lactylated proteins revealed that lactylated proteins were mainly enriched in pathways such as glycolysis/gluconeogenesis, the citrate cycle (TCA cycle), nitrogen metabolism, and the biosynthesis of nucleotide sugars (Fig. S1D, E). In the PPI network of the 4-VO+EA group, 386 nodes were identified; 366 proteins were upregulated, eight were downregulated, and 12 contained upregulated and downregulated sites (Fig. S1F) compared with the sham group. The lactylation-modified nodes primarily encompassed the following categories: citrate cycle (TCA cycle), endocrine and other factor-regulated calcium reabsorption, pentose phosphate pathway, long-term depression, and synaptic vesicle cycle.

~~
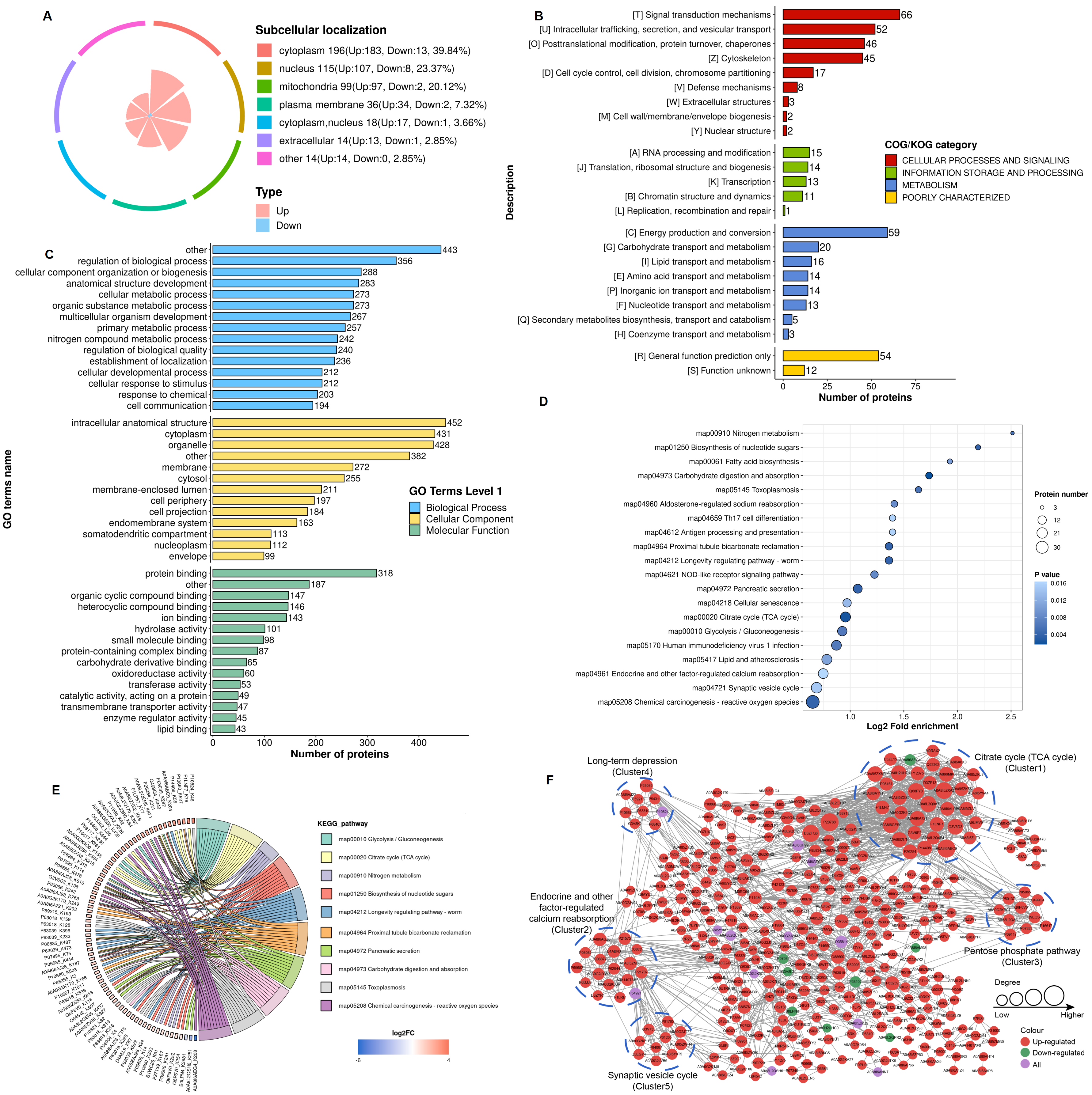
~~

**Figure S1. Functional classification and enrichment of lactylation modified proteins in the 4VO+EA and sham groups.** (**A**) Subcellular localization of lactylation modified proteins; (**B**) Regulated-kog classification; (**C**) Regulated-GO classification; (**D**) KEGG functional enrichment bubble chart; (**E**) KEGG functional enrichment chord diagram. 4-VO, four-vessel occlusion; EA, electroacupuncture; KEGG, Kyoto Encyclopedia of Genes and Genomes; GO, Gene Ontology; (**F**) The PPI network of lactylated proteins. 4-VO, four-vessel occlusion; KEGG, Kyoto Encyclopedia of Genes and Genomes; GO, Gene Ontology. PPI, protein-protein interaction.
