# Supplementary material for: Effect of electroacupuncture on hippocampal protein lactylation in a rat model of vascular dementia
Source: Front Neurol. 2025 Sep 2;16:1629474. doi: 10.3389/fneur.2025.1629474 (PMC12439496; doi:10.3389/fneur.2025.1629474)

**Table Comparison of Hippocampal Vdac3 Lactylation Levels Across Experimental Groups**

（$\bar{x}\pm s$, *n*=3）

| Group | Relative level of Vdac3 lactylation |
| --- | --- |
| Sham | 0.80±0.16 |
| 4-VO | 0.24±0.06^##^ |
| 4-VO+EA | 1.04±0.21^**^ |
| *F* | 61.527 |
| *P* | ＜0.001 |
| *P*1-value | ＜0.001 |
| *P*2-value | 0.003 |
| *P*3-value | ＜0.001 |

**Note:**Data are expressed as mean ± standard deviation. All variables satisfied the assumptions of normality (Shapiro-Wilk test, *P* > 0.05) and homogeneity of variance (Levene's test, *P* > 0.05). *P*1-values denote comparisons between Sham vs. 4-VO groups;*P*2-values denote comparisons between Sham vs. 4-VO+EA groups;*P*3-values denote comparisons between 4-VO vs. 4-VO+EA groups.^##^*P* < 0.01 versus Sham group.^**^*P* < 0.01 versus 4-VO group.

**IP and WB experiments detect Vdac3 three times repeated original pictures**

**Rep1：**

Kla


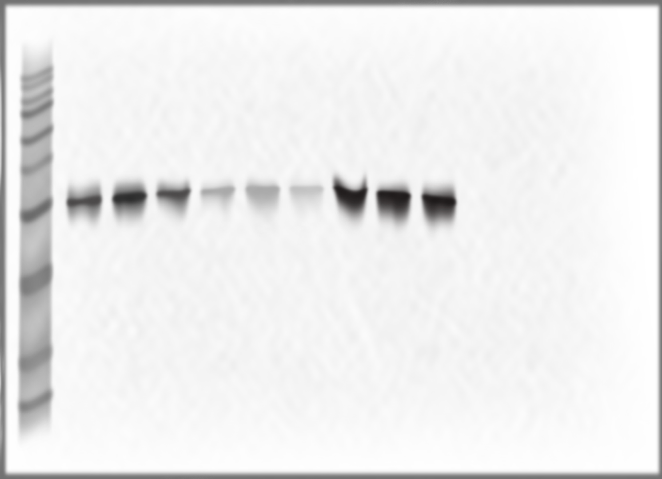


Vdac3


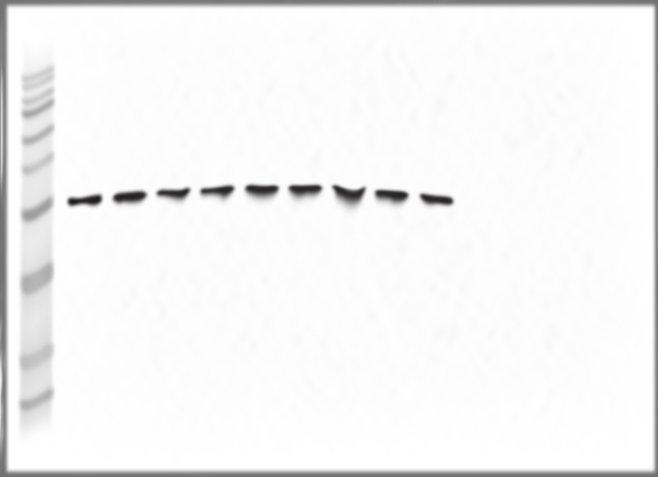


**Rep2：**

Kla


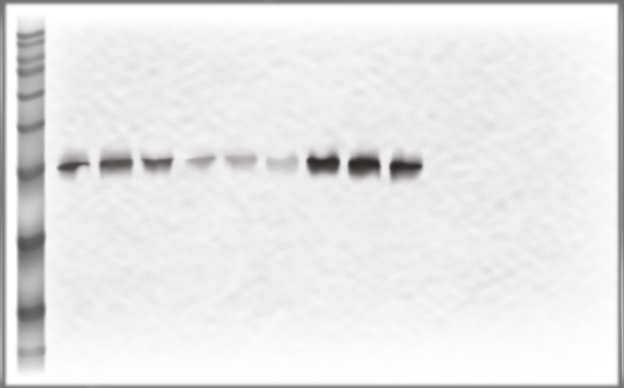


Vdac3


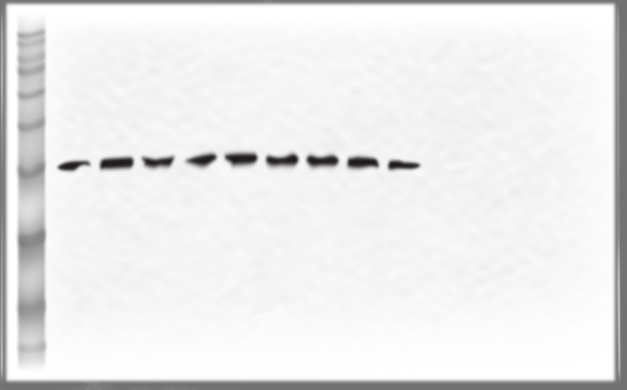


**Rep3：**

Kla


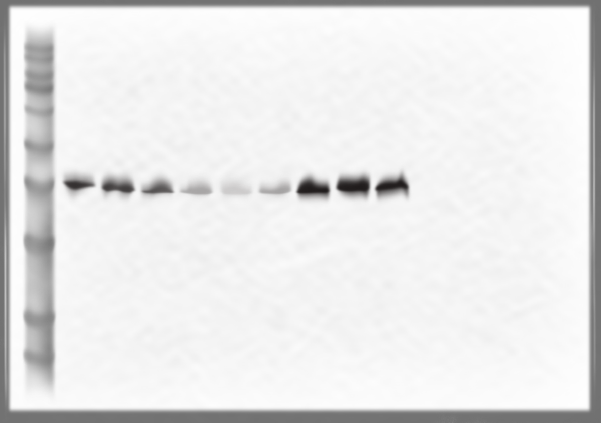


Vdac3


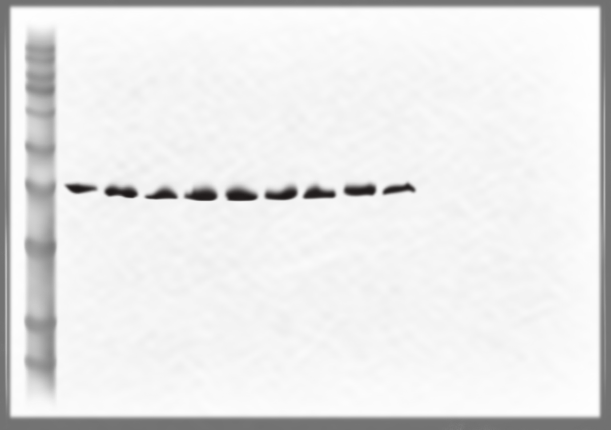

Supplement: Supplementary file 4 [file Data_Sheet_4.docx]
